# Metabolic rescue ameliorates mitochondrial encephalo‐cardiomyopathy in murine and human iPSC models of Leigh syndrome

**DOI:** 10.1002/ctm2.954

**Published:** 2022-07-25

**Authors:** Jin‐Young Yoon, Nastaran Daneshgar, Yi Chu, Biyi Chen, Marco Hefti, Ajit Vikram, Kaikobad Irani, Long‐Sheng Song, Charles Brenner, E. Dale Abel, Barry London, Dao‐Fu Dai

**Affiliations:** ^1^ Division of Cardiovascular Medicine, Department of Internal Medicine University of Iowa Carver College of Medicine Iowa City Iowa USA; ^2^ Abboud Cardiovascular Research Center, University of Iowa Carver College of Medicine Iowa City Iowa USA; ^3^ Department of Pathology, Carver College of Medicine University of Iowa Iowa City Iowa USA; ^4^ Department of Diabetes & Cancer Metabolism City of Hope National Medical Center Duarte California USA; ^5^ Fraternal Order of Eagles Diabetes Research Center and Division of Endocrinology and Metabolism, Department of Internal Medicine University of Iowa Carver College of Medicine, University of Iowa Iowa City Iowa USA

**Keywords:** Cardio‐encephalomyopathy, Leigh syndrome, mitochondria, Ndufs, nicotinamide riboside

## Abstract

**Background:**

Mice with deletion of complex I subunit Ndufs4 develop mitochondrial encephalomyopathy resembling Leigh syndrome (LS). The metabolic derangement and underlying mechanisms of cardio‐encephalomyopathy in LS remains incompletely understood.

**Methods:**

We performed echocardiography, electrophysiology, confocal microscopy, metabolic and molecular/morphometric analysis of the mice lacking Ndufs4. HEK293 cells, human iPS cells‐derived cardiomyocytes and neurons were used to determine the mechanistic role of mitochondrial complex I deficiency.

**Results:**

LS mice develop severe cardiac bradyarrhythmia and diastolic dysfunction. Human‐induced pluripotent stem cell‐derived cardiomyocytes (iPS‐CMs) with Ndufs4 deletion recapitulate LS cardiomyopathy. Mechanistically, we demonstrate a direct link between complex I deficiency, decreased intracellular (nicotinamide adenine dinucleotide) NAD^+^/NADH and bradyarrhythmia, mediated by hyperacetylation of the cardiac sodium channel Na_V_1.5, particularly at K1479 site. Neuronal apoptosis in the cerebellar and midbrain regions in LS mice was associated with hyperacetylation of p53 and activation of microglia. Targeted metabolomics revealed increases in several amino acids and citric acid cycle intermediates, likely due to impairment of NAD^+^‐dependent dehydrogenases, and a substantial decrease in reduced Glutathione (GSH). Metabolic rescue by nicotinamide riboside (NR) supplementation increased intracellular NAD^+^/ NADH, restored metabolic derangement, reversed protein hyperacetylation through NAD^+^‐dependent Sirtuin deacetylase, and ameliorated cardiomyopathic phenotypes, concomitant with improvement of Na_V_1.5 current and SERCA2a function measured by Ca2^+^‐transients. NR also attenuated neuronal apoptosis and microglial activation in the LS brain and human iPS‐derived neurons with Ndufs4 deletion.

**Conclusions:**

Our study reveals direct mechanistic explanations of the observed cardiac bradyarrhythmia, diastolic dysfunction and neuronal apoptosis in mouse and human induced pluripotent stem cells (iPSC) models of LS.

## Graphical abstract

- Leigh syndrome (LS) is a severe mitochondrial encephalomyopathy that may involve heart.
- Its metabolic derangement is characterized by NAD^+^ deficiency and impairment of several NAD^+^‐dependent metabolic enzymes and Sirtuins.
- Nicotinamide Riboside restores Sirt1 function, reverses hyperacetylation of several crucial proteins, including Nav1.5 and SERCA2 in cardiomyocytes, and p53 in the brain, leading to amelioration of bradycardia, neuronal loss and encephalopathy.

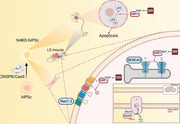

## 1 INTRODUCTION

Leigh syndrome (LS) is a severe mitochondrial disorder that manifests as psychomotor regression early in life,^1^ and treatment options are very limited, as is the case for many mitochondrial disorders. The most common causes are mutations in components of mitochondrial complex I, NADH dehydrogenase, which oxidises NADH to (nicotinamide adenine dinucleotide) NAD^+^. It consists of 45 subunits, one of which is NADH dehydrogenase [ubiquinone] iron–sulphur protein 4 (Ndufs4). There is a wide spectrum variant of LS caused by mutations of different subunits of mitochondrial complex I^2^ or other mitochondrial respiratory complexes. The complexity of LS genetics and protean clinical manifestations impose great challenges to develop mouse models to study LS. A few mouse models have been developed, such as mice with Ndufs4^1^, ^3^ or Ndufs6 deletion.^4^, ^5^ Mice with a homozygous germline deletion of exon 2 of the encoding gene (*Ndufs4^−^* ^/^ *^−^*) exhibit phenotypes resembling the severe encephalomyopathy in LS patients.^6^ These include features of growth retardation, lethargy, loss of motor skills, ataxia, hypothermia, slowed breathing and apnea. The latter has been shown to contribute to early death (∼45–60 days after birth) in this mouse model.

Among LS patients, approximately 18%–21% have cardiac involvement and this is associated with a worse prognosis. Cardiac abnormalities in LS may include cardiomyopathy, pericardial effusion, and conduction abnormalities. Hypertrophic cardiomyopathy has been reported as the most common abnormality in these patients.^7^, ^8^ In Ndufs4^−/−^ mouse models, while the encephalomyopathy is unequivocal, cardiac involvement remains debatable. In two independent studies describing mice with cardiac‐specific Ndufs4 deletion, one showed hypertrophic cardiomyopathy,^3^ while the other reported normal cardiac function.^9^

In the present study, we sought to elucidate the metabolic derangement and underlying mechanisms of cardio‐encephalomyopathy in LS using germline Ndufs4^−/−^ mice *(also called LS mice*). We found that these mice develop severe bradyarrhythmia and diastolic dysfunction related to hyperacetylation of the cardiac sodium channel (Na_V_1.5) and the calcium handling protein sarco/endoplasmic reticulum Ca^2+^‐ATPase 2a (SERCA2a), respectively. To date, there is no effective treatment for LS other than supportive therapy with bicarbonate to manage lactic acidosis and various vitamin B supplements. Niacin (nicotinic acid), nicotinamide and nicotinamide riboside (NR) are members of vitamin B3 family that function as “salvageable precursors” to replenish intracellular NAD^+^ via 2–3 metabolic steps. Among members of vitamin B3 family, NR has the best pharmacokinetics profiles compared with nicotinic acid and nicotinamide, as an NAD^+^ supply to improve health in metabolic stress mouse models.^10^ In a state of NAD^+^ deficiency, as in LS mice, niacin will be consumed to replenish NAD^+^, thus substantially depleted. Using daily supplementation of NR,^11^ we discovered that NR increased intracellular NAD^+^, reversed protein hyperacetylation, ameliorated metabolic derangement, restored sinus rhythm, normalised diastolic function, ameliorated the decreases in Na_V_1.5 current and restored SERCA2 function in LS mice and human‐induced pluripotent stem cells derived cardiomyocytes (hiPS‐CMs) with homozygous Ndufs4 deletion. We further demonstrated that NR attenuates acetyl‐p53‐mediated neuronal apoptosis in LS mouse brain (cerebellum and midbrain) and iPS‐derived mixed neurons with the Ndufs4 deletion. These data provide a mechanistic explanation for the pathophysiology of LS cardio‐encephalomyopathy and its underlying metabolic derangement.

## 2 RESULTS

### 2.1 Metabolic derangement in LS indicates decreased function of several NAD^+^‐dependent enzymes, which is partially restored by nicotinamide riboside

**FIGURE 1:**
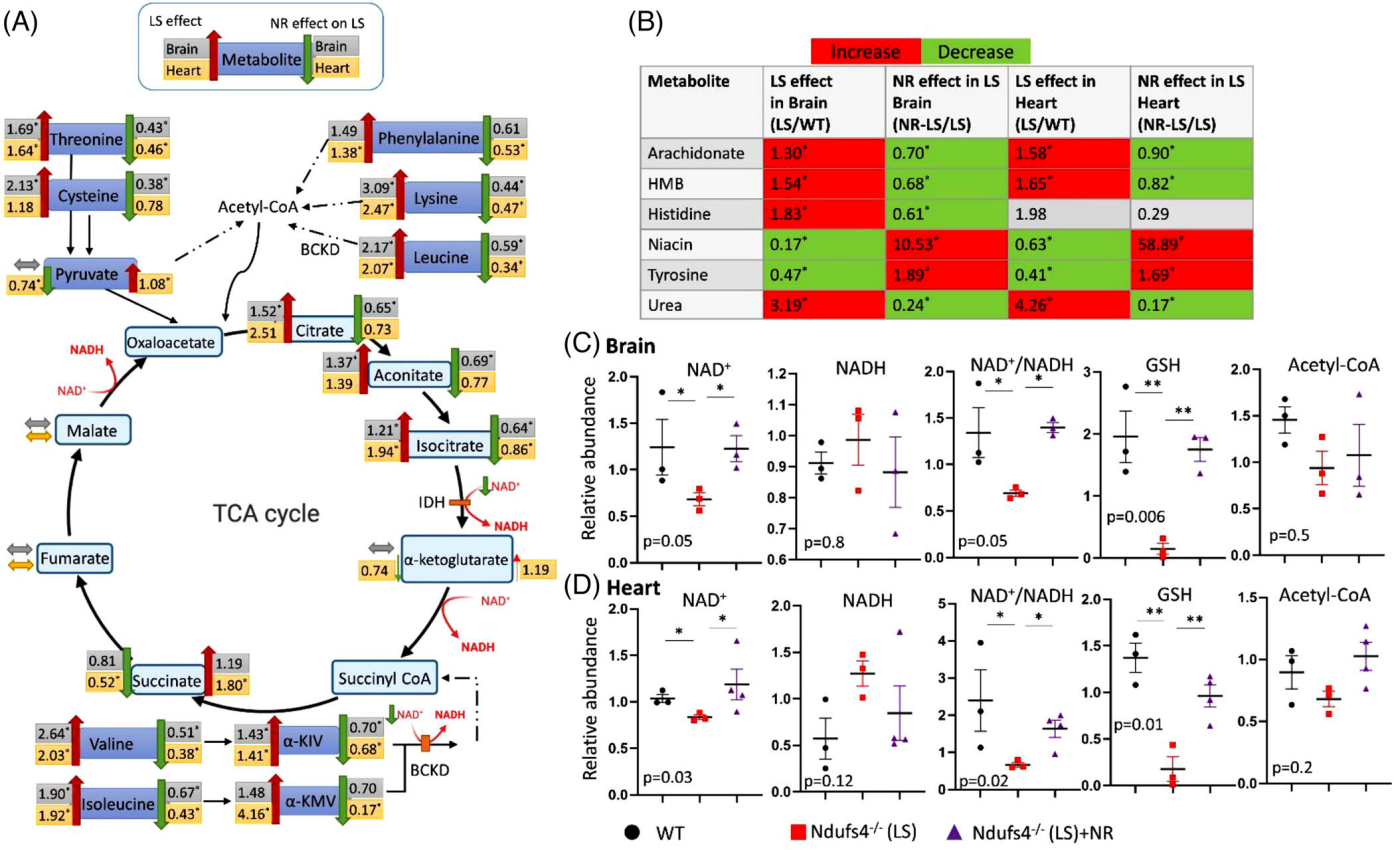
Metabolomic derangement in Leigh syndrome (LS) mouse brain (cerebellum/brainstem) and heart, and the effect of nicotinamide riboside. (A) Ratios of several metabolites measured by gas chromatography and mass spectrometry in brain and heart of Ndufs4^−/−^ (LS) vs. WT (indicating LS effect, left, *n* = 4) and NR‐treated Ndufs4^−/−^ (LS) vs. untreated LS (indicating NR effect, right, *n* = 3). IDH: Isocitrate dehydrogenase, BCKD: branched‐chain α‐keto acid dehydrogenase, α‐KIV: ketoisovalerate, α‐KMV: α‐ keto‐β‐methylvaleric acid. (B) Relative abundance of significantly changed metabolites (*) in the indicated groups. HMB: beta‐Hydroxy‐beta‐Methylbutyric‐acid (Table S1–S3). (C) Brain (cerebellum and brainstem) and (D) heart metabolites data measured by liquid chromatography /mass spectrometry in LS mice with or without NR treatment. Data are mean ± s.e.m. of biologically independent samples. The listed *p*‐values were determined by Kruskal–Wallis test; *n* = 3–4 each group. *p < .05, **p < .01 were calculated by non‐parametric tests. Note: the small‐sample size maybe under‐powered to detect small changes of some metabolites.

We performed multiple‐targeted metabolomics analyses to investigate changes of several metabolites in the LS heart and brainstem/cerebellum. We focused on brainstem and cerebellum since these regions have the most prominent brain pathologies and can be quickly dissected and flash‐frozen to preserve tissue metabolites. The metabolomic dataset is presented in Table S1 (1st, WT vs. LS hearts), Table S2 (2nd, WT vs. LS vs. NR‐treated LS hearts), Table S3 (2nd, WT vs. LS vs. NR‐treated LS brainstem and cerebella) and is summarised in Figure 1A,B. Since NADH dehydrogenase deficiency in LS impairs the oxidation of NADH to NAD^+^ causing decreased NAD^+^ (particularly in mitochondria), the metabolic profiles of LS brain and hearts are mainly characterised by derangement of several NAD^+^‐dependent metabolic enzymes. The most prominent findings were significant increases in many amino acids (ranged from +36% to > 3‐fold increase in both hearts and brains), urea and metabolic derivatives of branched‐chain amino acid (BCAA) aminotransferase, including α‐keto‐isocaproate and β hydroxy‐β‐methyl‐butyric acid (both from leucine), α‐ketoisovalerate (KIV, from valine) and α‐keto‐β‐methylvaleric acid (KMV, from isoleucine). These α‐keto‐derivatives of BCAA usually undergo oxidation by branched‐chain α‐keto acid dehydrogenases (BCKD), key enzymes requiring NAD^+^ as a cofactor. BCKD enzymes were likely impaired because of decreased NAD^+^/NADH in LS hearts and brains. There were accumulation of some tricarboxylic acid (TCA) cycle intermediates, including citrate, aconitate, and isocitrate, which is likely related to impaired activity of the NAD^+^‐dependent isocitrate dehydrogenase. Arachidonate, a critical signalling molecule and a precursor of various eicosanoids (such as prostaglandins, etc.), was increased. There were decreases in niacin (a precursor of NAD^+^) and tyrosine. NR supplementation (500 mg/kg/day, i.p.) significantly reversed and restored many of these metabolomics changes associated with Ndufs4 deficiency in both cerebellum/brainstem and heart (Figure 1A,B). There were no significant or consistent changes in glycolytic, pentose phosphate pathways, purine, or pyrimidine metabolites.

Using liquid chromatography followed by mass spectrometry analysis of these samples, we demonstrated that Ndufs4 deletion in LS (which results in the deficiency of NADH dehydrogenase) led to decreased NAD^+^, NAD^+^/NADH, dramatic depletion of the reduced form of glutathione (GSH), a major intracellular antioxidant system, in LS hearts and brains (Figure 1C,D), but there was no significant change in acetyl‐CoA. NR supplementation significantly rescued NAD^+^/ NADH and restored GSH levels in LS hearts and brains (Figure 1C,D), thereby ameliorated many metabolic defects due to NAD^+^ deficiency and redox stress (Figure 1A,B).

### 2.2 Ndufs4^−/−^ mice (LS mice) were runted and had cardiomyopathy

**FIGURE 2:**
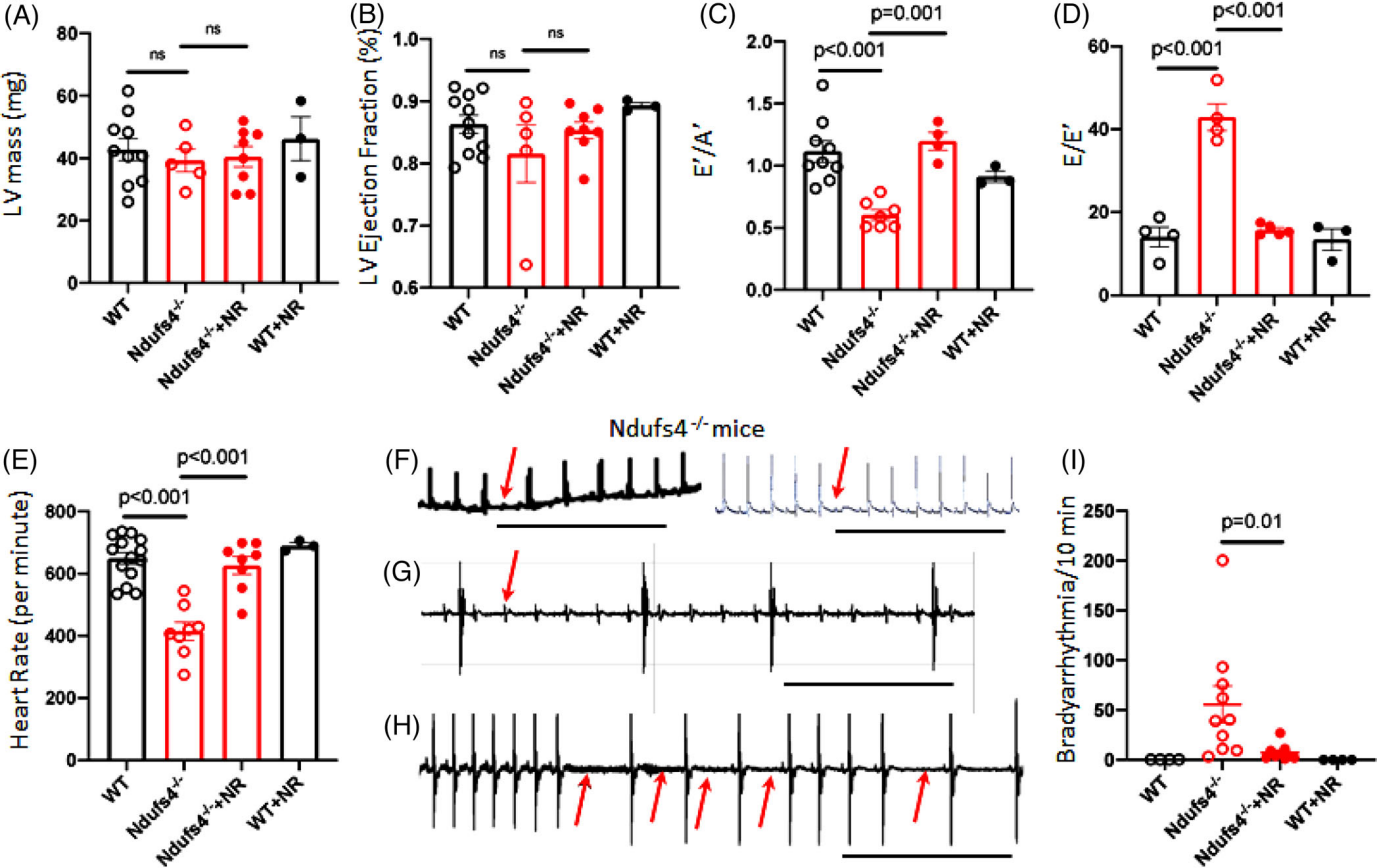
Diastolic dysfunction and bradyarrhythmia in Ndufs4 deficient mice is reversed by treatment with nicotinamide riboside (NR). Echocardiographic measurements of (A) left ventricle mass (LV), (B) LV ejection fraction, (C) E’/A’ determined by tissue Doppler imaging (impaired diastolic function defined as E`/A` < 1), (D) E/E’, (E) heart rate (*n* = 3–10). (F–H) Representative ECG of Ndufs4^−/−^ mice, showing (F) episodic second‐degree atrioventricular (AV) block, (G) third‐degree AV block, and (H) sinus arrhythmia. Scale bar: 1 sec. (I) Quantitation of arrhythmic events over a 10‐minute period in wild type, Ndufs4^−/−^ (LS) mice, with or without NR treatment (*n* = 4–9). ns, not significant. Data are mean ± s.e.m. Statistical significance was determined by ANOVA, followed by post hoc analysis.

Echocardiography of conscious mice showed that left ventricle (LV) mass and ejection fraction were normal in LS mice (Figure 2A,B), but tissue Doppler imaging of LS mice showed that diastolic function was impaired (Figure 2C,D), as evidenced by decreased E`/A` ratio of relaxation velocity of mitral annulus during early diastole (E’) to late diastole (A’), and increased E/E`. In addition, these mice had severe bradycardia, with a median resting heart rate (HR) of ∼400 bpm (Figure 2E), reflecting a ∼35% decrease from the baseline physiological HR of > 600 bpm. Both diastolic dysfunction and bradycardia were significantly ameliorated by supplementation with NR (500 mg/kg/day, i.p.) (Figure 2C–E). LS mice were runted, with their average body weight ∼60% that of wild‐type (WT) littermates, and NR supplementation did not alter this (Figure S1a). Heart weight and heart weight normalised to tibia length did not differ significantly from that in WT mice, and this was not affected by NR supplementation (Figure S1B,C). Pathological analysis assessing ventricular size and fibrosis did not distinguish between hearts from WT mice and their LS counterparts with or without NR treatment (Figure S1D–G).

Electrocardiograms (ECGs) from conscious immobilised LS mice demonstrate a spectrum of bradyarrhythmia. These include occasional second‐degree atrioventricular (AV) block (red arrow, Figure 2F), rare high‐grade AV block (Figure 2G), and frequent sinus node dysfunction (Figure 2H). Bradyarrhythmia were frequently recorded in LS mice (Figure 2I) but was absent in WT control or NR‐treated WT mice. Supplementation with NR substantially reduced arrhythmic events in LS mice (Figure 2I).

### 2.3 Hyperacetylation of Na_V_1.5 promote bradyarrhythmia in LS mice

**FIGURE 3:**
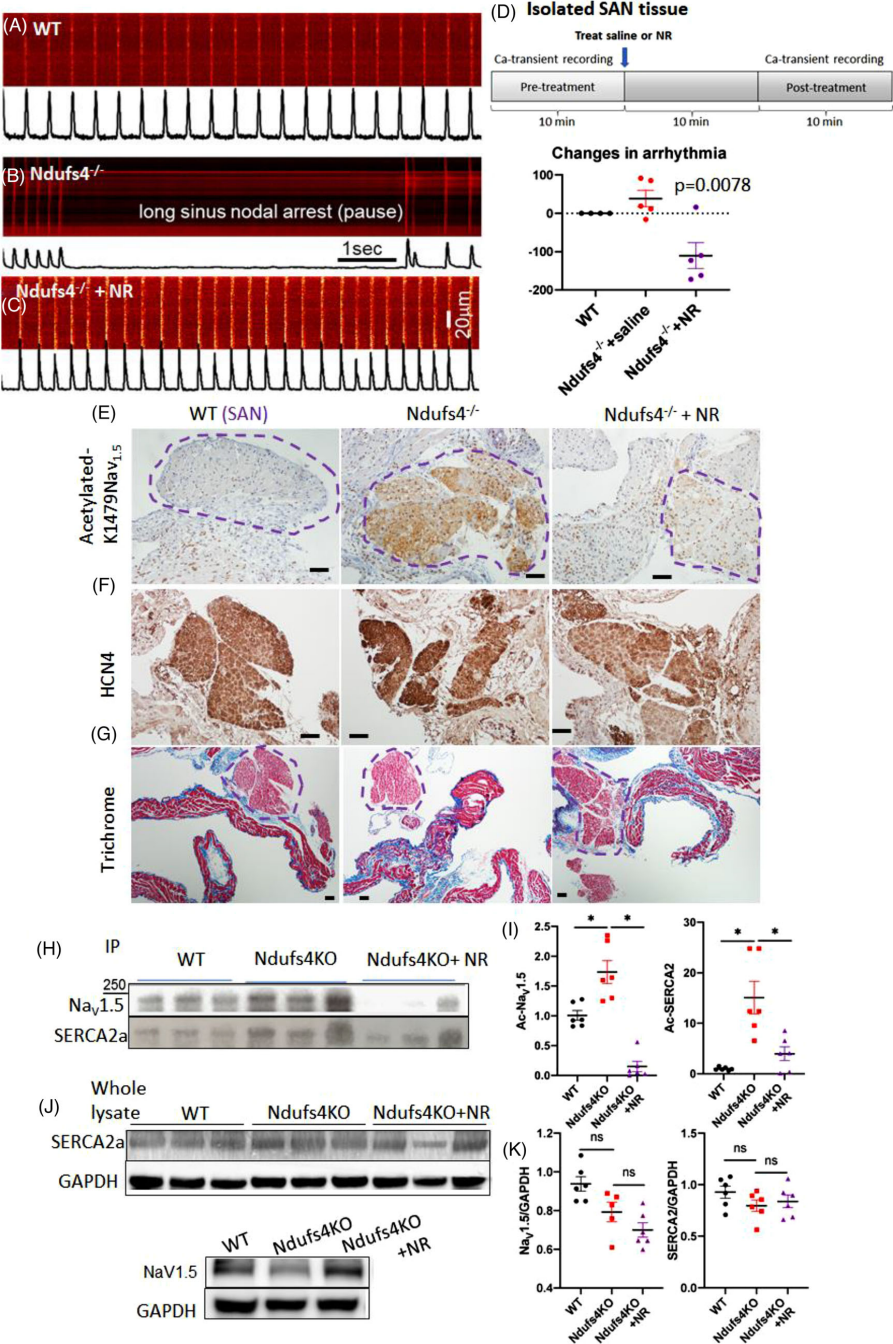
Characteristics and mechanism of bradyarrhythmia in Ndufs4^−/−^ Leigh syndrome () mice. (A–C) Analyses of sinoatrial node (SAN) tissue dissected from the hearts of mice from three designated groups. Confocal imaging of Ca^2+^‐transients in SANs tissue from (A) WT, (B) Ndufs4^−/−^ and (C) Ndufs4^−/−^ mice treated with nicotinamide riboside (NR). (D) Quantitation of changes in arrhythmic events in SANs over a 10‐min period (post‐pretreatment) (*p* = .03 using Kruskal–Wallis). (E) Immunohistochemical staining of SANs with specific antibody against acetylated K1479‐ Na_V_1.5 in WT, Ndufs4^−/−^, and NR‐treated Ndufs4^−/−^ tissue. (F) Immunohistochemical staining of HCN4 in heart tissue sections of WT, Ndufs4^−/−,^ and NR‐treated Ndufs4^−/−^ to highlight SAN (encircled in E and G). (G) Trichrome staining of SANs in WT, Ndufs4^−/−^, and NR‐treated Ndufs4^−/−^ mice tissues. (H,I) Western blot of mouse heart proteins from specified treatment groups following immunoprecipitation with total acetyl‐lysine antibodies and probing with Na_V_1.5 or SERCA2a antibodies and quantifications (*n* = 6 per group). (J,K) Total Na_V_1.5 and SERCA2a proteins in mouse hearts of the indicated experimental groups and quantification (*n* = 5–6 per group) (*p < .05, ns not significant). Data are mean ± s.e.m. of biologically independent samples. Statistical significance was determined by ANOVA, followed by post hoc analysis

To exclude autonomic dysfunction or metabolic acidosis as potential contributors to the bradyarrhythmia observed in Ndufs4^−/−^ (LS) mice, we dissected sinoatrial nodal (SAN) tissue, loaded it with the Rhod‐2 calcium indicator dye ex vivo, and assessed calcium transients by confocal microscopy. In Ndufs4^−/−^ SAN tissue, there were multiple long pauses in spontaneous [Ca^2+^] transients, suggesting SA nodal arrests (Figure 3B). Intermittent pauses, marked bradycardia with or without episodic tachycardia (bradycardia–tachycardia) representing various spectrum of sinus node dysfunction were observed in Ndufs4^−/−^ SAN (Figure S2A). NR supplementation significantly reduced the bradycardic events and restored sinus rhythm (Figure 3C,D) and Ca^2+^‐transient amplitudes similar to those recorded from WT SAN samples (Figure 3A).

To address whether cell‐autonomous mechanism is sufficient to cause arrhythmia, we crossed Ndufs4^flox/flox^ mice with HCN4‐Cre mice to generate conduction tissue‐specific deletion of Ndufs4. Approximately 2–3 months after tamoxifen‐induced deletion of Ndufs4, 87.5% of the HCN4–Ndufs4^−/−^ mice developed various forms of arrhythmia, including atrial or ventricular premature contractions, sinus bradycardia and frequent sinus pauses (Figure S2B) resembling those found in germline Ndufs4^−/−^ LS mice (Figure 2). This finding supports the hypothesis that Ndufs4 deficiency in the cardiac conduction system itself is sufficient to cause arrhythmia in a cell‐autonomous manner, although the metabolic defect of Ndufs4^−/−^ in neurons, cardiomyocytes, and other cell types could also contribute to arrhythmia by impacting the neurohormonal or paracrine regulation of cardiac rhythm.

Ndufs4 deletion led to reduced NAD^+^/NADH (Figure 1C), a change that can impair the function of NAD^+^‐dependent enzymes, including Sirt1.^12^ To identify the potential mechanisms of arrhythmia and diastolic dysfunction in LS mice, we examined two critical cardiac proteins: sodium channel Na_V_1.5 and SERCA2a.^13^ Na_V_1.5 governs the initiation and propagation of cardiac action potential, whereas SERCA2a is the key protein regulating the decay of calcium transients by reuptake into sarcoplasmic reticulum. Both proteins are reported to be modulated by Sirt1.^14^, ^15^ We have previously shown that acetylation of the K1479 residue in Na_V_1.5 impairs the trafficking of this channel protein, causing a decrease in the inward depolarising Na^+^ current (*I*_Na_) conducted by this channel. To examine the role of acetylation in LS hearts, we performed immunohistochemistry using an antibody specific for acetylated Na_V_1.5 at K1479 and showed that K1479 in Na_V_1.5 was significantly hyperacetylated in the LS mice SAN (Figure 3E). HCN4 immunostaining highlighted SAN and the staining patterns overlap with that of Ac‐K1479Na_V_1.5 (Figure 3E,F). Trichrome stain did not show any obvious fibrosis within SAN (Figure 3G). Hyperacetylated K1479‐ Na_V_1.5 was also noted in LV sections from LS hearts (Figure S3A,B). NR supplementation dramatically reversed this acetylation in both SAN and LV in LS mouse hearts (Figure 3E, Figure S3C), concomitant with the observed amelioration of arrhythmia and restoration of the normal sinus rhythm (Figure 3A–C). For quantitative analysis, we performed immunoprecipitation with acetyl‐lysine antibody and demonstrated that the acetylated forms of Na_V_1.5 and SERCA2a were substantially increased in LS hearts. The acetylated Na_V_1.5 and SERCA2a were significantly attenuated by NR supplementation (Figure 3H,I). The hyperacetylation was not specific to these proteins, as there was an increase in global protein acetylation levels in the hearts of LS mice, and this was also attenuated by NR treatment (Figure S3D). In addition to its functional impairment by hyperacetylation, Na_V_1.5 protein abundance slightly decreased in LS mice (not significant), and this may also contribute to bradycardia in LS mice. NR supplementation did not affect the total Na_V_1.5 levels. There was no significant change of the SERCA2a protein abundance among groups (Figure 3J,K, Figure S3E,F).

### 2.4 Acetylation of K1479 of Na_V_1.5 in mitochondrial complex I‐deficient cells decreases the inward depolarising Na^+^ current

**FIGURE 4:**
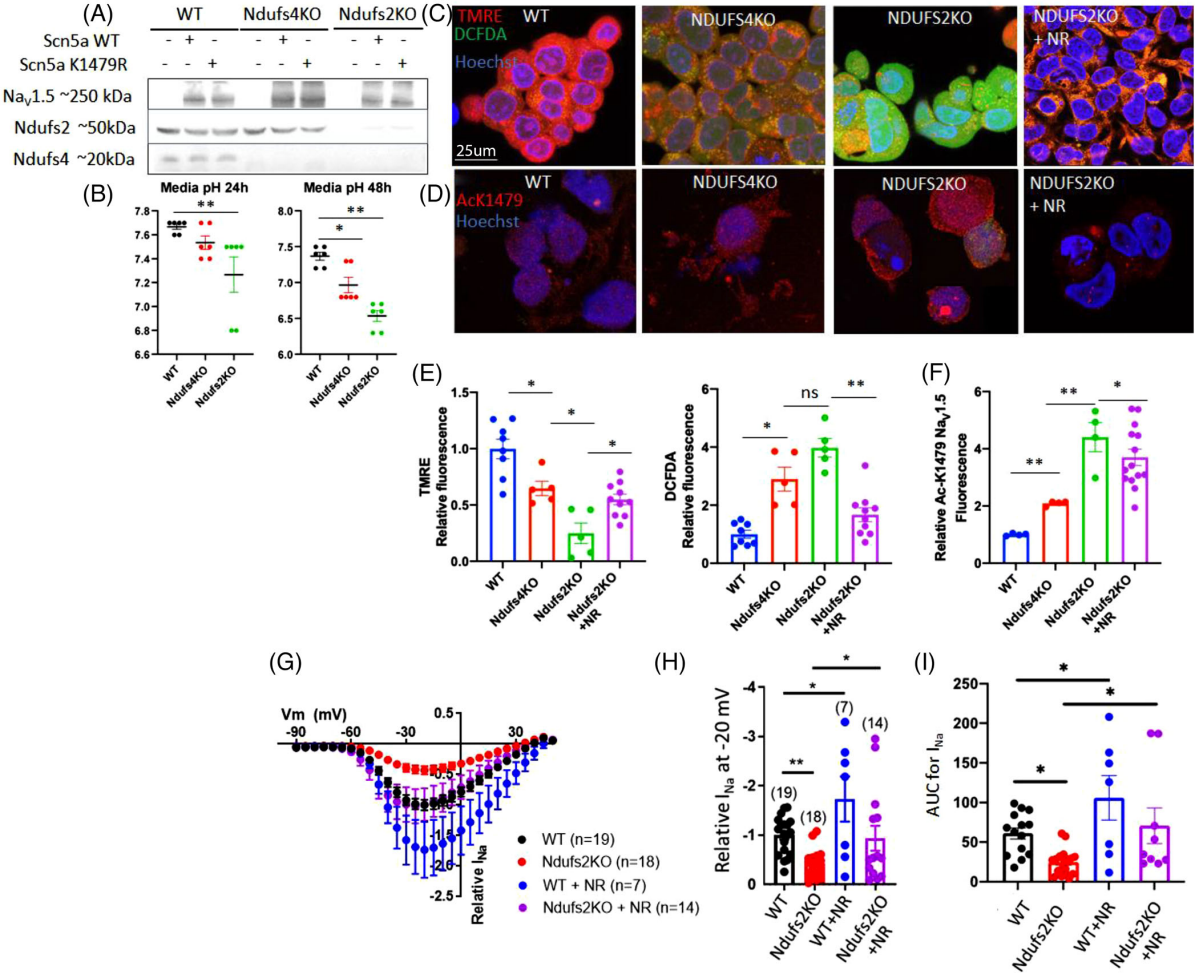
Acetylation of K‐1479‐ Na_V_1.5 in mitochondrial complex I‐deficient cells decrease sodium current. (A) Western blot for Na_V_1.5 protein in 293 cells lacking Ndufs4 (Ndufs4 KO) or Ndufs2 (Ndufs2 KO) which were transfected with plasmid carrying WT or mutant Scn5a gene (to produce WT or acetylation‐site mutant Na_V_1.5 proteins). (B) pH of conditioned‐media from 293, Ndufs4 KO, and Ndufs2 KO cells at 24 or 48 h after plating (*n* = 6/group). (C) Live staining of 293 (WT), Ndufs4 KO, and Ndufs2 KO cells with markers of mitochondrial membrane potential (TMRE), total cellular ROS (DCFDA) and Hoechst (blue). (D) Representative fluorescence images of acetylation of Na_V_1.5 residue K1479. (E) Quantitation of results in panel C (*n* = 5–10/group). (F) Quantitation of results in (D). (*n* = 4–12/group). (G) Patch clamp results of *I*_Na_ for WT and Ndufs2 KO cells with or without NR treatment. Quantitation of (H) relative *I*_Na_ density at −20 mV and (I) AUC for the indicated groups in panel (G). *n* = 7–19. Data are mean ± s.e.m. of biologically independent samples. Statistical significance was determined by ANOVA and post‐hoc analyses. *p < .05, **p < .01, ns not significant

To further investigate the role of K1479‐Na_V_1.5 acetylation in the context of mitochondrial complex I deficiency, we used HEK293 cells heterologous expression system. Since the endogenous Na_V_1.5 expression in HEK293 is negligible, we introduced exogenous WT or K1479 mutant, as confirmed by Western blots (Figure 4A). NDUFS4 and NDUFS2 knock‐out (KO) HEK 293 cells were generated by CRISPR/Cas9 method, followed by transfection with WT or K1479 mutant‐Na_V_1.5 (Figure 4A). Interestingly, the deletion of NDUFS2 also resulted in a substantial decrease in NDUFS4 expression. As mitochondrial complex I is a large complex and NDUFS2 is located adjacent to the junction of the inner membrane and matrix domain, we proposed that its loss destabilised the matrix domain leading to degradation of the NDUFS4 subunit. The loss of both subunits in the NDUFS2 KO cells resulted in a more acidic phenotype than the NDUFS4 KO cells, as shown by the conditioned‐media pH. At 24 h, the NDUFS2 KO cells developed a significantly more acidic media (pH ∼7.2) than WT cells (pH ∼7.7, *p* = .023). At 48 h, the media pH from NDUFS4 KO cells dropped to ∼6.9 (vs. pH ∼7.3 in WT, *p* = .01), but it remained less acidic than NDUFS2 KO media (pH ∼6.5, *p* = .006, Figure 4B). In addition to increased acid production, NDUFS2 KO cells also had increased global protein acetylation (Figure S4A).

Live‐cell staining with a marker of mitochondrial membrane potential, tetramethylrhodamine ethyl ester (TMRE), and a marker of reactive oxygen species (ROS), 2′,7′‐dichlorodihydrofluorescein diacetate (DCFDA), showed reduced TMRE in NDUFS4 KO cells, indicating that mitochondrial membrane potential was decreased. Conversely, DCFDA fluorescence was increased, suggesting that total cellular ROS levels were higher (Figure 4C,E). NDUFS2 KO cells displayed a much greater loss of mitochondrial membrane potential and a greater increase in cellular ROS than the NDUFS4 KO cells, and this was significantly ameliorated upon NR supplementation (Figure 4C,E). Citrate synthase activity, a surrogate marker of mitochondrial content, showed no significant change in NDUFS4 KO and NDUFS2 KO cell lines compared with WT 293 cells (Figure S4B). To elucidate the role of SIRT1 in NR‐treated cells, we knocked down SIRT1 in NDUFS2KO cell line using SIRT1 siRNA. SIRT1 knockdown abolished the effect of NR, indicating that the beneficial effect of NR was at least partly mediated through SIRT1 (Fig S4C,D).

Immunofluorescence staining with an antibody specific for acetylated K1479‐Na_V_1.5 revealed a ∼2‐fold increase in AcK1479‐Na_V_1.5 in NDUFS4 KO cells and a ∼4‐fold increase in NDUFS2 KO cells, which was significantly decreased after NR supplementation (Figure 4D,F). To determine the functional relevance of Na_V_1.5 hyperacetylation on this residue, we performed a patch‐clamp experiment on the WT and NDUFS2 KO cells, which have more severe phenotypes than NDUFS4 KO cells. The inward depolarising Na^+^ current (*I*_Na_) was significantly lower in the NDUFS2 KO cells (Figure 4G–I), as shown by the *I*_Na_ at −20 mV and the area under the current–voltage curves (AUC). Pre‐treatment with NR for 24 h significantly restored *I*_Na_ in NDUFS2 KO cells and potentiated the *I*_Na_ of WT control cells (Figure 4G–I).

To further investigate the importance of K1479‐Na_V_1.5 acetylation on the Na^+^‐current phenotype of Ndufs2KO cells, we transfected WT HEK293 cells with a lysine to glutamine mutant construct, K1479Q‐Na_V_1.5, that mimics constitutively acetylated K1479 Na_V_1.5. (The molecular structure and charge of glutamine resembles acetyl‐lysine). We showed decreased *I*_Na_ in K1479Q^14^ (acetylation mimic) that was not rescued by NR (Figure S5A,B). Interestingly, when the K1479Q‐Na_V_1.5 was expressed in NDUFS2 KO cells, no further decrease in *I*_Na_ was observed compared with WT Na_V_1.5 in these NDUFS2 KO cells and NR had no significant effect (Figure S4C,D). These findings suggest that Na_V_1.5 acetylation mimic and NDUFS2 KO cells share a similar mechanism in decreasing *I*_Na_. Taken together, these results support the hypothesis that acetylation of Na_V_1.5 at K1479 contributes to decreased *I*_Na_ induced by NDUFS2 deletion and that deacetylation by NR at K1479 enhances *I*_Na_. Next, we generated an acetylation‐deficient mutant construct, K1479R‐Na_V_1.5 (Arg (R) resembles Lysine (K) in term of charge at physiological pH and molecular structure but is not acetylable—mimicking a persistent deacetylation state). K1479R‐Na_V_1.5 mutant significantly increased *I*_Na_ in both WT and NDUFS2 KO cells (Figure S5E,F). Neither NDUFS2KO nor NR treatment affected *I*_Na_ conducted by the deacetylation mimic K1479R‐Na_V_1.5 in NDUFS2 KO cells (Figure S5E,F). Restoration of *I*_Na_ in K1479R‐Na_V_1.5 NDUFS2KO cells and the absence of NR effect on *I*_Na_ in this deacetylation mimic mutant suggest that acetylation of K1479R‐Na_V_1.5 likely mediates the decreased *I*_Na_ in NDUFS2 KO cells, and the beneficial effect of NR is at least partly mediated by deacetylation of K1479R‐Na_V_1.5. To elucidate the role of Sirt1, we treated HEK293 cells expressing WT Na_V_1.5 with the Sirt1 inhibitor, Ex‐527.^14^, ^16^ Our data showed that Ex‐527 abolish the NR‐mediated enhancement of *I*_Na_ (Figure S5G,H), suggesting that the NR effect is mediated, at least in part, by the NAD^+^‐dependent Sirt1 deacetylation of Na_V_1.5. Direct measurement of Sirt1 activity showed decreased Sirt1 activity in NDUFS4 KO hearts (*p* = .12) and cerebellar tissues (*p* = .07) and it was partially restored by NR (Figure S6). Taken together, our data suggest that Na_V_1.5 acetylation is one of the mechanisms leading to *I*_Na_ decrease in NDUFS2 KO and the NR beneficial effect in NDUFS2 KO is mediated via Sirt1 deacetylation of Na_V_1.5.

### 2.5 Deletion of NDUFS44 decreased Na_V_1.5 current in human iPS‐derived cardiomyocytes

**FIGURE 5:**
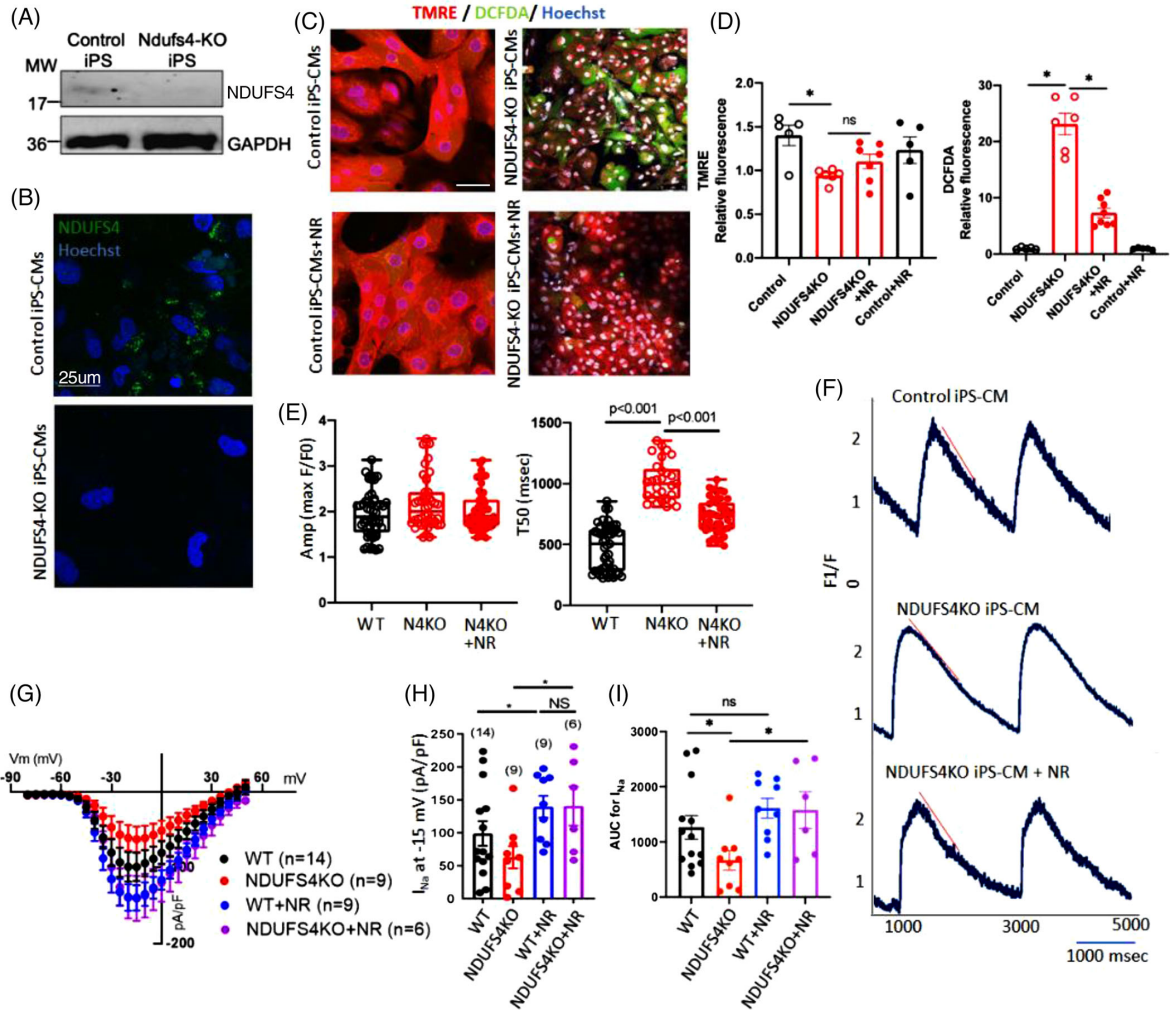
Deletion of Ndufs4 Decreased Na_V_1.5 current in human iPS‐derived cardiomyocytes. (A) Representative immunoblots and (B) immunofluorescence images of WT and Ndufs4 KO human iPS‐CMs immunolabelled for Ndufs4. (C) Live staining of control and Ndufs4 KO human iPS‐CMs, with or without NR pretreatment, with TMRE (red), DCFDA (green), and Hoechst (blue). (D) Quantitation of fluorescence in panel (C). (Data representative of three independent experiments) (*n* = 5–8 per group). (E,F) Amplitude, decay rate (T50) and calcium transient in control and Ndufs4 KO human iPS‐CMs with or without NR treatment, red line representing the slope (*N* = 20 per group). (G) *I*_Na_–voltage curves of WT and Ndufs4KO human iPS‐CMs with or without NR treatment (1 mM). *n* = 6–14. Quantification of *I*_Na_ density at –15 mV (H) and AUC (I) for the indicated groups in panel (G). ANOVA and post hoc analyses; *p < .05, NS not significant

To increase the translational relevance of our findings in mice and heterologous cells (HEK293), we created NDUFS4 deletion using the CRISPR/Cas9 method in human iPS cells generated from a healthy Caucasian male (ATCC‐1026). The deletion of NDUFS4 was confirmed by Western blot and immunostaining of the iPS‐Cardiomyocytes (iPS‐CMs) (Figure 5A,B) at ∼30 days post‐differentiation under tri‐iodo‐thyronine (T3)‐supplemented media to enhance cardiomyocytes maturation.^17^ Troponin staining was used as a marker of cardiomyocytes to confirm successful cardiomyocyte differentiation from induced pluripotent stem cells (iPSCs) and co‐staining of troponin and Ndufs4 display the scattered NDUFS4 interspersed among striated pattern of Troponin in iPS‐cardiomyocytes (Figure S9). We performed live‐cell staining of isogenic control (WT) and NDUFS4 KO iPS‐CMs. Compared with control iPS‐CM, NDUFS4 KO cells had much decreased TMRE staining (loss of red) and elevated DCFDA staining (increased green), and this was mitigated by pre‐treatment with NR (1 mM) for 24 h (Figure 5C,D). These findings suggest that NDUFS4 KO cardiomyocytes have a lower mitochondrial membrane potential and higher levels of cellular ROS than WT control (Figure 5C,D), and that pre‐treatment with NR mitigates these effects. We measured Ca^2+^ transients in these iPS‐CMs (paced at 0.5 Hz) and found that NDUFS4 KO cells had a substantially slower decay rate (longer T50, time to 50% from peak) compared with isogenic iPS‐CM control (*p* < .001) and this was significantly improved by NR treatment (*p* < .001, Figure 5E [right panel] and 5F). The amplitude of Ca^2+^ transients was not significantly different among all three groups (Figure 5E [left panel] and 5F). These findings suggest a slower SERCA2 Ca^2+^ reuptake in NDUFS4 KO iPS‐CM that was significantly ameliorated by NR treatment. To confirm the findings in HEK293 cells, we performed patch‐clamp to measure the inward depolarising Na^+^ current (*I*_Na_) in NDUFS4 KO iPS‐CMs and its isogenic WT control. As shown by the current–voltage curves (Figure 5G), *I*_Na_ density at −15 mV (Figure 5H) and area under the curve (AUC) of *I*_Na_–voltage curves (Figure 5I), NDUFS4 knock‐out reduced *I*_Na_ by ∼45% (*p* = .088 for *I*_Na_ at −15 mV and *p* = .04 for AUC of current–voltage curves, Figure 5H,I). Pre‐treatment with NR for 24 h significantly enhanced *I*_Na_, especially in the NDUFS4 KO iPS‐CMs (Figure 5G–I).

### 2.6 NR ameliorates LS mice activity, neuronal apoptosis, and microgliosis

**FIGURE 6:**
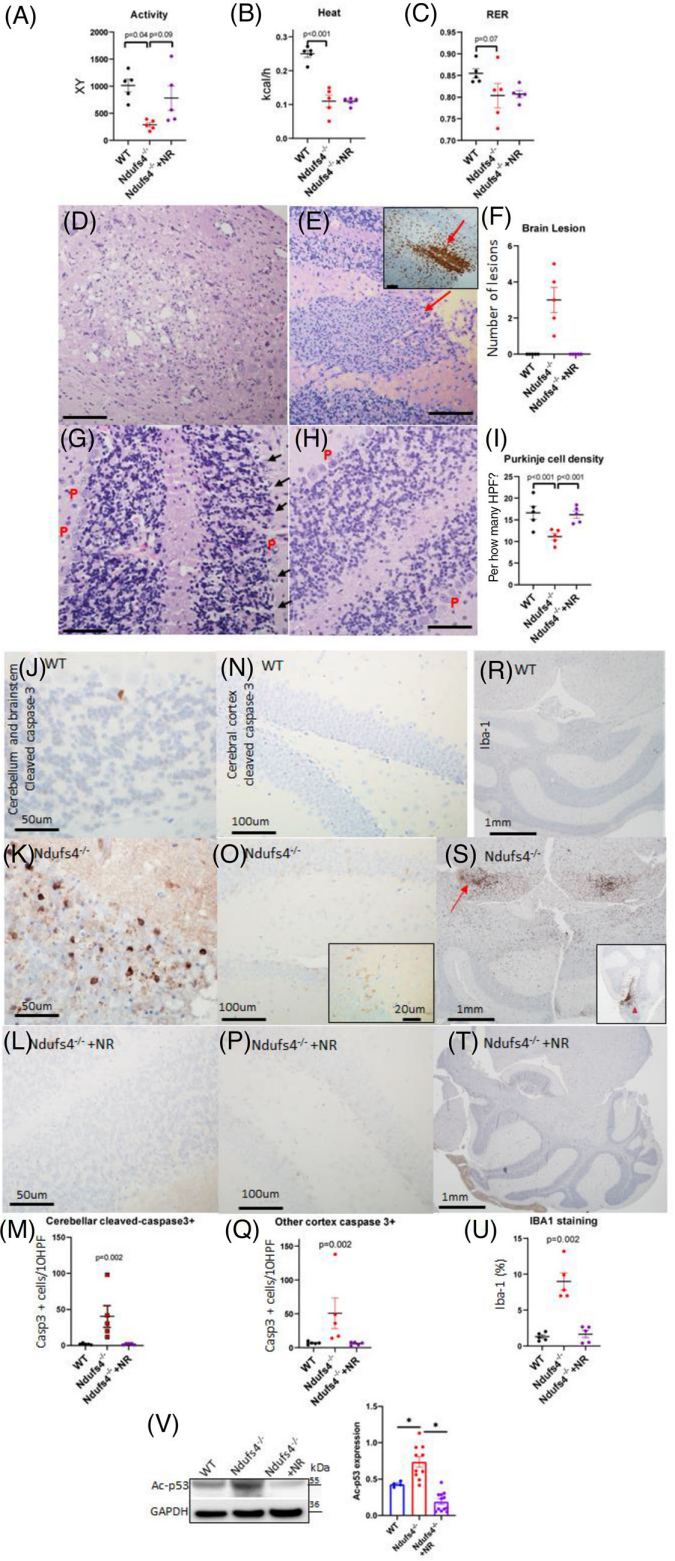
Metabolic derangement and neuropathology in Leigh syndrome (LS) mice. (A–C) Measurement of (A) mouse activity, (B) heat produced, and (C) respiratory exchange ratio (RER) using metabolic chambers; *n* = 5–6. Neuropathology of Ndufs4^−/−^ brain as detected by H&E staining of: (D) brainstem region, showing vacuolisation of neuropils (spongiosis); (E) cerebellum, showing microglial and vascular proliferation (red arrow) (Iba‐1 expression in the inset); (F) quantification of lesions described in (D,E); (G) cerebellar cortex, showing Purkinje (P) cells (arrow). (H) Neuropathology of Ndufs4^−/−^ cerebellum following treatment with NR, as detected by H&E staining showing Purkinje cell preservation. (I) Quantification of Purkinje cell density in all indicated groups. For all panels shown, Scale bar: 100 μm, *n* = 4–5. (j–m) Cerebellum and brainstem stained for activated caspase‐3 per 10 high power fields (40×) (HPFs), for (J) WT, (K) Ndufs4^−/−^ and (L) NR‐treated Ndufs4^−/−^ mice, (M) quantitation of caspase‐3 positive cells. (N–Q) Cerebral cortex stained for activated caspase 3, and quantitation in mice of the same genotypes as in panels (J)–(L). Inset in (O) is higher magnification image. (R)–(U) Iba‐1 expression in the dorsal midbrain (*s*, arrow) and cerebellar cortex (*s*, arrowhead in the inset) of (R) WT, (S) Ndufs4^−/−^ and (T) NR‐treated Ndufs4^−/−^ mice, (U) quantitation of Iba‐1 immunostaining; *n* = 5–6; Kruskal–Wallis tests for (M)–(U). (V) Representative western blot of K120‐p53 in mouse cerebellum/brainstem from specified treatment groups and quantification (*n* = 4–12). Data are mean ± s.e.m. Statistical significance was determined by ANOVA post hoc (*p < .0001)

LS mice had impaired motor function, characterised by cerebellar ataxia, and decreased locomotor activity, as measured by CLAMS metabolic chambers (Figure 6A). Energy expenditure (heat production) and respiratory exchange ratio (RER) were lower in LS versus WT mice (Figure 6B,C). The changes in energy expenditure and RER were not significantly affected by NR. However, NR supplementation significantly ameliorated the ataxia (see Video S1–S3) and improved daily locomotor activity (Figure 6A). Neuropathological examination of the LS brain demonstrated spongiosis, defined as edema of neuropils (Figure 6D), and proliferation of microglia and vascular cells (Figure 6E, red arrow), particularly in the cerebellum and the brainstem region. Semiquantitative analysis of hematoxylin and eosin (H&E)‐stained LS brain sections by a neuropathologist blinded to treatment group revealed increased foci of these lesions in the LS mice but none in NR‐treated LS counterparts (Figure 6F). Quantitative analysis of Purkinje cell density in the cerebellar cortex showed a significant reduction of Purkinje cells in the LS mice, indicating that neurons were lost (Figure 6G,I), and this was attenuated by NR treatment (Figure 6H,I).

Immunohistochemistry for cleaved‐caspase‐3 revealed prominent apoptosis among cells of the Purkinje layer and the underlying granular neurons in the cerebellum of untreated LS mice (Figure 6K,M). Apoptotic cells were scattered throughout the cerebral cortex in LS mice (Figure 6O,Q). Apoptosis was associated with prominent activation of microglia, as the number of Iba‐1 positive microglia in the dorsal midbrain (Figure 6S) and cerebellar cortex (Figure 6S, inset) were significantly higher in the LS mice. Treatment with NR significantly mitigated neuronal apoptosis in both the cerebral cortex and dorsal midbrain (Figure 6L,M,P,Q), and the associated activation of microglia in the LS brain (Figure 6T,U). Given that acetylation of p53 has been shown to enhance apoptosis,^18^ we performed Western blots of acetylated‐p53 in cerebellar/brainstem lysates. Acetylated p53 at K382 and K120 residues in brain lysates from Ndufs4^−/−^ mice were increased compared with those from WT mice (Figure 6V, Figure S7A–C). NR supplementation significantly reduced the hyperacetylation of p53, concomitant with the amelioration of neuronal apoptosis (Figure 6J–Q). Also, quantitative PCR analysis of some p53 target genes, including *Aen*, *Bax* and *Traf4*, showed increased expression of all three target genes in Ndufs4KO brain compared with WT and treatment with NR reduced the expression (Figure S7D), providing a confirmation of p53 activation / acetylation. Consistent with neuroprotective and cardioprotective effects, NR significantly extended the lifespan of LS mice (Figure S7E).

Interestingly, although Ndufs4^−/−^ promoted apoptosis in the susceptible neurons of the cerebellar and brainstem regions, it did not induce apoptosis in cardiac left ventricular tissue (Figure S8A–C). In line with the absence of apoptosis in cardiac left ventricular tissue of Ndufs4KO mice, we did not observe any change in Sirt1 expression and p53 acetylation in the heart tissue of untreated or NR treated Ndufs4KO mice compared with WT (Figure S8D,E).

### 2.7 Increased p53 acetylation and apoptosis in the Ndufs4‐deficient brain

Our finding of enhanced apoptosis in the cerebellum and brainstem of Ndufs4^−/−^ mice and that this effect was ameliorated by NR supplementation (Figure 5J–U) suggested that protein hyperacetylation plays a critical role in apoptosis in the susceptible brain regions.

**FIGURE 7:**
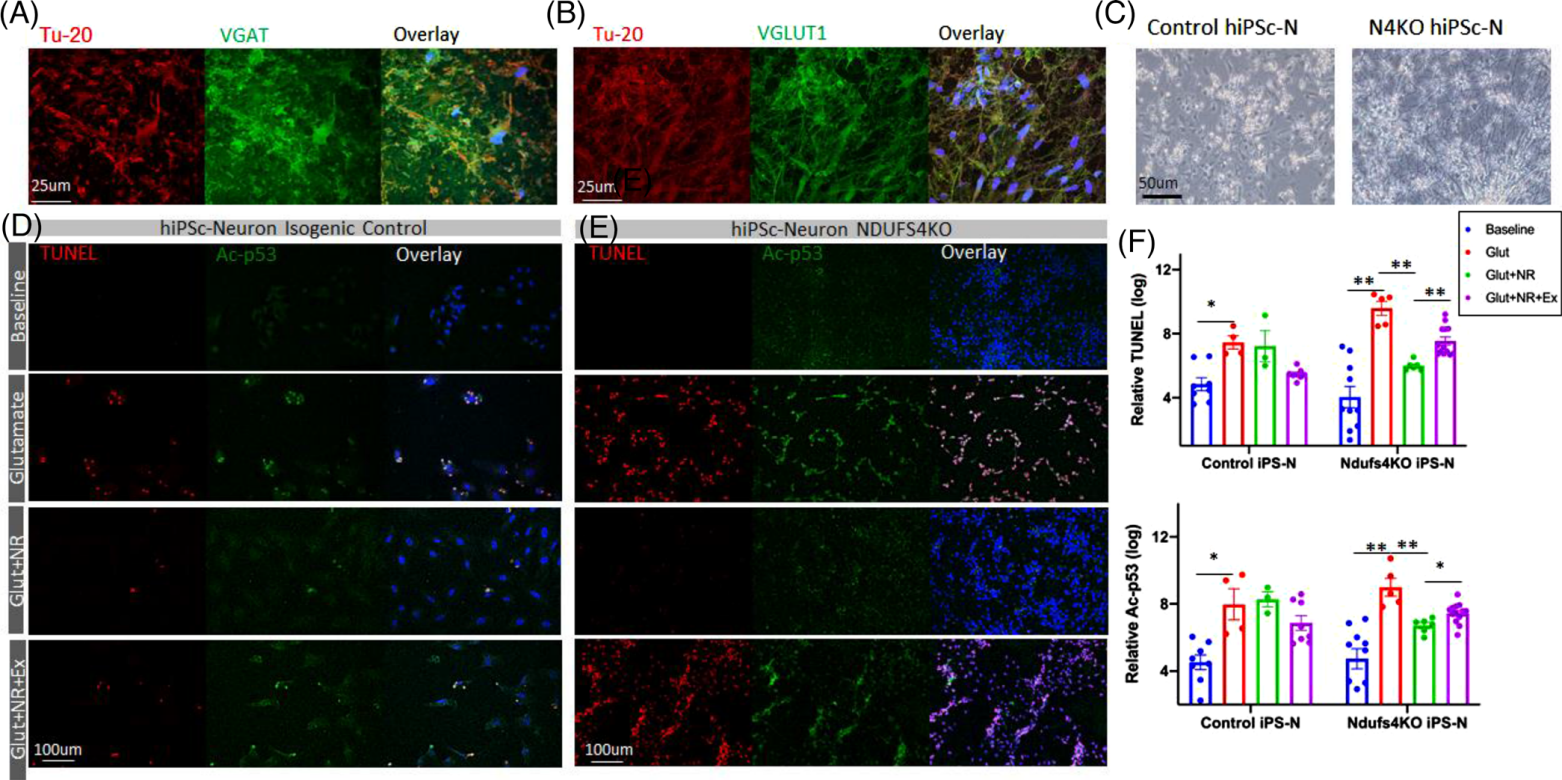
Human iPS‐Neurons with Ndufs4KO are more susceptible to glutamate‐induced apoptosis, mediated through acetylation of p53. (A,B) Characterisation of human iPS‐Ns by immunofluorescence for pan‐neuronal marker β‐Tubulin (Tu‐20, red), vGAT (vesicular GABA transporter, green) or vGluT1 (vesicular glutamate transporter, green) and Hoechst (blue). (C) Morphology of mature human iPS‐neurons. (D,E) Representative fluorescence images of control and Ndufs4KO iPS‐neurons treated with glutamate (30 μM), NR (1 mM), and Ex‐527 (10 μM). (F) Quantitation of relative fluorescence intensity of indicated groups in (D,E) (log‐transformed for data normalisation). Data are mean ± s.e.m *p < .01, **p < .001, by ANOVA post hoc tests

To further confirm this, we examined iPS‐derived neurons (iPS‐N) for impaired Sirt1‐dependent deacetylation of p53, as a potential mechanism of neuronal apoptosis. NDUFS4 knock‐out iPS and isogenic WT iPS controls were differentiated into neuronal progenitor cells (NPCs), which were then stimulated to form neural rosettes using embryoid‐body‐based method (STEMCELL). The NPCs were allowed to mature into a mixed population of cells expressing a marker of GABAergic (the vesicular GABA transporter, VGAT in green, Figure 7A) or glutamatergic (vesicular glutamate transporter 1, VGluT1, in green, Figure 7B) neurons. These VGAT or VGluT1 markers colocalised with the pan‐neuronal marker beta‐3 tubulin, Tu‐20 (Figure 7A,B, red), indicating a mixed population of iPS‐Ns. At ∼30 days post‐differentiation from neural rosettes (Figure 7C), these mixed iPS‐Ns were stimulated with glutamate (30 μM), with or without NR (1 mM). The effect of glutamatergic excitotoxicity after 24 h was assessed by TUNEL assay and measurement of acetyl‐p53 (Figure 7D,E). Acetylation of p53 at either lysine K120 or K382 promotes p53‐dependent apoptosis^18^, ^19^ in response to DNA damage, cell stress, and oncogenic stress.^20^ Glutamate excitotoxicity led to a significant induction of apoptosis (TUNEL staining) in control iPS‐Ns, in parallel with an increase in p53 acetylation (*p* = .003). NR treatment with or without Ex‐527, an inhibitor of Sirt1, in control iPS‐Ns stimulated with glutamate did not significantly alter TUNEL or acetylated p53 staining (Figure 7D,F). In contrast, the glutamate excitotoxicity was potentiated in NDUFS4KO iPS‐Ns, shown by substantial increases in both TUNEL and Ac‐p53 in response to glutamate stimulation (*p* < .001, Figure 7E,F). Treatment with NR significantly attenuated p53 acetylation and TUNEL positivity in response to glutamate toxicity in the NDUFS4KO iPS‐N cells. Simultaneous treatment with Ex‐527 and NR significantly abolished the protective effect of NR on glutamate toxicity in NDUFS4KO iPS‐N cells. The partial inhibition of NR beneficial effect by Ex‐527 suggests that the anti‐apoptotic effect is mediated, at least in part, by NR‐dependent Sirt1 deacetylation of target proteins, including p53.

## 3 DISCUSSION

This study demonstrates novel mechanisms underlying cardio‐encephalomyopathy in LS. Our analysis of mouse and iPS‐CM models of this disease revealed that hyperacetylation of Na_V_1.5 promotes bradyarrhythmia and hyperacetylation of SERCA2a contributes to diastolic dysfunction. We further showed that the reversal of hyperacetylation by NR supplementation ameliorates these cardiomyocyte abnormalities in the context of Ndufs4 deficiency, in vivo and in vitro. Our study also provides a novel mechanistic explanation for the loss of neurons in LS, with involvement of p53 hyperacetylation and increased apoptosis in cerebellar and brainstem regions. Using targeted metabolomics, we demonstrated metabolic alterations in LS hearts and cerebellum/brainstem, characterised by significant increase in many amino acids and their metabolic derivatives, arachidonate and accumulation of TCA cycle intermediates (citrate, aconitate and isocitrate), decreased glutathione, niacin, and NAD^+^. We further showed that NR restores intracellular NAD^+^, glutathione, and many of these metabolic changes. NR also attenuated p53 acetylation in iPS‐Ns and the cerebellum and brainstem of LS mice, leading to an attenuation of neuronal apoptosis and mitigation of neuropathological lesions in LS brain.

Previous studies using germline LS mice focused largely on encephalomyopathy phenotypes.^6^ However, in clinical settings, ∼18%–21% of LS patients have cardiac pathology, including hypertrophic cardiomyopathy and/or conduction abnormalities.^7^, ^8^ Thus, it is important to understand the cardiac manifestations in LS mice, which are not well characterised. Previous studies have utilised various muscle‐specific loss of Ndufs4 to elucidate the role of mitochondrial complex I in cardiac physiology and metabolism. Previous study reported that mice with myocyte‐specific loss of Ndufs4 (driven by CKM‐NLS‐Cre)^3^ had hypertrophic cardiomyopathy. In contrast, mice with cardiomyocyte‐specific loss of Ndufs4 (driven by αMHC) did not show significant structural abnormalities or systolic dysfunction but was found to have aggravated pressure‐overload induced heart failure, and this was associated with increased protein hyperacetylation due to alterations in the redox state and inhibition of Sirt3 activity.^9^ However, a more recent study from the same group showed that ex vivo ischemia‐reperfusion injury was ameliorated in these αMHC‐Ndufs4^−/−^ mice, with a reduction of ROS, suggesting that ROS arising during reperfusion injury is generated mainly by complex I.^21^

**FIGURE 8:**
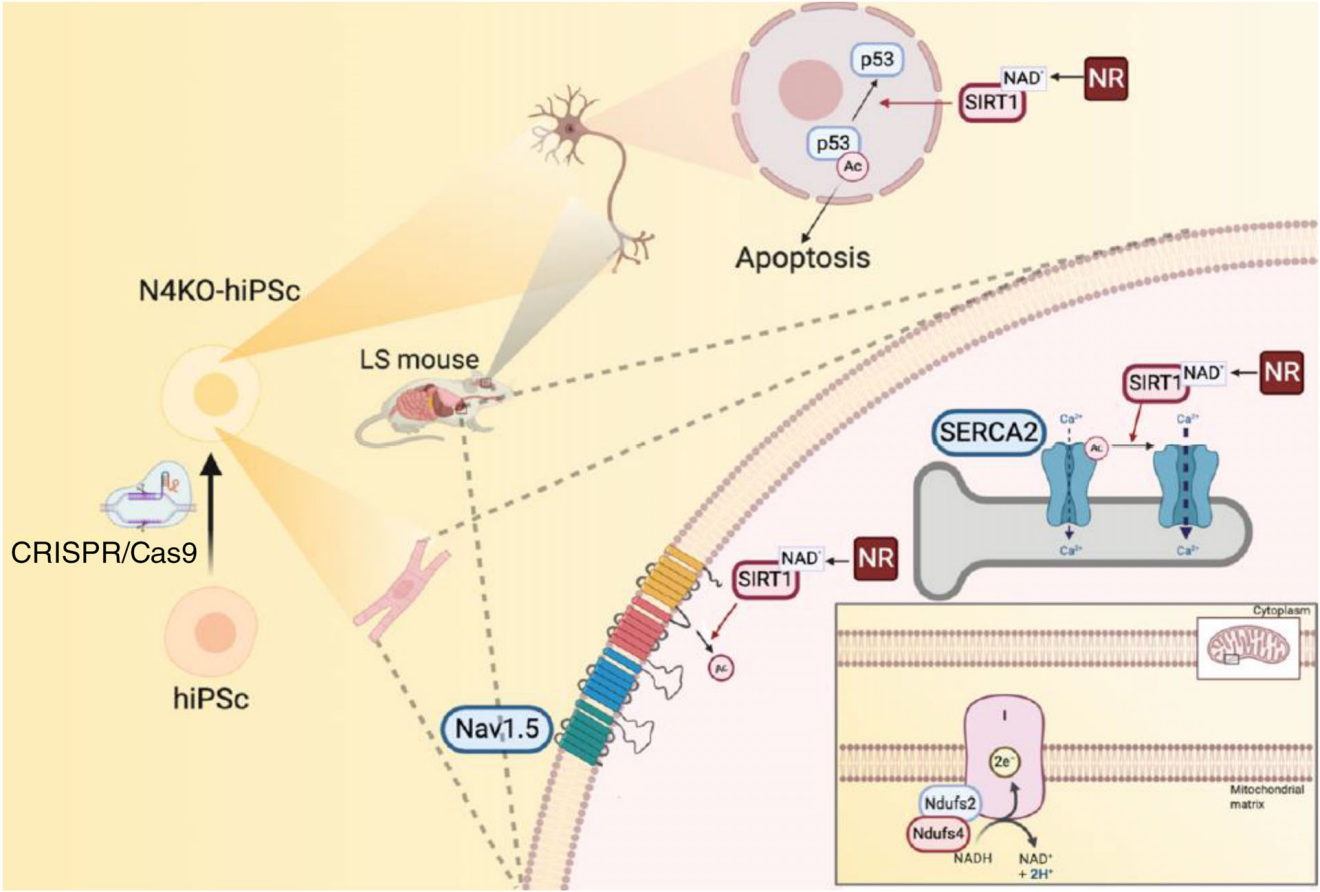
Schematic illustration of the molecular mechanism of cardio‐encephalomyopathy in Ndufs4 KO mice and hiPSCs (created by Biorender).

The current study elucidates novel mechanisms contributing to several phenotypes of cardio‐encephalomyopathy in LS mice. First, we reported a novel finding of severe bradyarrhythmia, including SAN dysfunction (sick sinus syndrome) and occasional heart block that were associated with hyperacetylation of the K1479 in Na_V_1.5. Na_V_1.5 is expressed in the ventricular myocardium^22^ and sinoatrial node in which it is involved in the propagation of the action potential.^23^ Deletion of NDUFS subunits in heterologous system (HEK293) and iPS‐CMs impaired various NAD^+^‐dependent sirtuin deacetylases.^14^ Our previous study showed that membrane trafficking of Na_V_1.5 is disrupted by hyperacetylation of the K1479 residue in Na_V_1.5 in Sirt1^–/–^ mice, and this led to reduced *I*_Na_ and impaired electrical activity in the heart.^14^ This study is the first to link mitochondrial complex I deficiency and the mechanisms underlying bradyarrhythmia with reduced NAD^+^/NADH, which leads to hyperacetylation of K1479 in Na_V_1.5 and subsequent reduction of the *I*_Na_, resembling the conduction block seen in MHC‐Sirt1^–/–^ mice. The fact that NR reversed hyperacetylation of Na_V_1.5 at K1479 and enhanced *I*_Na_ in concert with restoration of normal sinus rhythm suggests that the hyperacetylation of Na_V_1.5 leads to bradyarrhythmia in the context of Ndufs4 deletion in LS hearts (Figure 8). In addition to Sirt1‐dependent Na_V_1.5 acetylation, previous studies showed an acetylation‐independent increase in *I*_Na_ by NR supplementation through PKC phosphorylation of Na_V_1.5.^15^, ^24^ Furthermore, a defect in mitochondrial complex I may also trigger redox stress,^25^ which has been shown to slow Na_V_1.5 inactivation, and/or to decrease the peak *I*_Na_, leading to arrhythmia.^26^, ^27^ Our mass spectrometric analysis in LS mouse hearts showed substantial reductions in reduced glutathione (GSH), a major reducing substrate in the endogenous antioxidant system, which was also restored by NR supplementation, suggesting that the beneficial effect of NR may also be mediated, at least in part, by restoring GSH (Figure 1C,D).

Second, LS mice exhibited significant diastolic dysfunction in association with hyperacetylation of SERCA2a, despite the absence of hypertrophic cardiomyopathy or systolic dysfunction. Hyperacetylation of SERCA2a was recently shown to reduce Ca^2+^ reuptake into SR, indicating SERCA2a dysfunction.^28^ Increased SERCA2a acetylation had been documented in various failing heart samples, including end‐stage human cardiac explants, murine hearts with pressure‐overload induced heart failure, and porcine models of myocardial infarction. These observations suggest that hyperacetylation of SERCA2a might be a shared mechanism leading to impaired SERCA2a function in failing hearts.^28^ As NR reversed the hyperacetylation status and improved the function of SERCA2a in LS mouse hearts and NDUFS4‐deficient iPS‐CMs, our data suggest that SERCA2a hyperacetylation, which affects its Ca^2+^ reuptake function, may contribute to diastolic dysfunction in LS mice.

Third, LS‐encephalomyopathy showed a substantial loss of neurons, increased neuronal apoptosis and increased infiltration of the cerebellum and brainstem by activated microglia, concomitant with an increase in acetylated‐p53 and overall increased inflammatory phenotype reported in Ndufs4 deficient mice.^29^ Acetylation of p53 has been shown to mediate activation of the downstream effector p21, leading to apoptosis.^20^ In this study, the beneficial effects of NR in decreasing acetylated‐p53, neuronal apoptosis (both in vitro and in vivo), attenuating microglial activation and overall neuropathology in the LS mouse brain suggests that activation/hyperacetylation of the p53‐mediated apoptotic pathway contributes to the severe encephalopathy observed in LS mice (Figure 8). Mechanistically, Sirt1‐mediated deacetylation of p53 inactivates the sequence‐specific transcriptional activity of the protein and represses p53‐mediated arrest of cell growth and apoptosis in response to DNA damage and oxidative stress. This process also prevented p53‐dependent transactivation of p21.^30^ Several lysines in human p53 (K120, K370, K372, K373, K381, K382) are acetylated following various forms of DNA damage, enhancing the transcriptional activity of p53 and regulating the fate of the cell. As we have shown, acetylation of p53 at K120, within its DNA‐binding domain, is crucial for p53‐mediated apoptosis. Our findings are reinforced by a previous study demonstrating that Sirt1 plays a neuroprotective role in the context of brain ischemia through deacetylation and subsequent inhibition of p53‐induced and nuclear factor κB‐induced apoptotic and inflammatory pathways.^31^ Similarly, the Sirt1‐mediated brain‐protective effect of Maresin1 (macrophage mediator in resolving inflammation) in a mouse model subjected to middle cerebral artery occlusion demonstrates the involvement of Sirt1 signalling in the reduction of mitochondrial damage in the context of cerebral ischemia reperfusion injury.^32^

The role of protein hyperacetylation in heart failure has been controversial. Increased global, mitochondrial, and metabolic protein acetylation has been documented in αMHC‐Ndufs4^−/−^ mouse hearts, human heart failure and several experimental models, suggesting protein hyperacetylation is critical in the pathogenesis of heart failure. In contrast, a recent study showed that extreme acetylation of the cardiac mitochondrial proteome in carnitine acetyltransferase and Sirt3 double knock‐out (DKO) mice did not potentiate pressure‐overload‐induced heart failure. Even though the mitochondrial acetyl‐lysine landscape of these DKO hearts was elevated to levels much higher than those observed in response to pressure overload or Sirt3 KO alone, deep phenotyping of mitochondrial function in the DKO revealed a surprisingly normal bioenergetics profile.^33^ The current study provides a direct mechanistic link between mitochondrial dysfunction and cardiomyopathic phenotypes caused by impairment of Sirt1 due to NAD^+^ deficiency. Our study of LS mice elucidates mechanisms underlying bradyarrhythmia and diastolic dysfunction which is promoted by hyperacetylation of Na_V_1.5 and SERCA2a, respectively. Both Na_V_1.5 and SERCA2a are critical cardiac proteins and well‐known targets of Sirt1, rather than the targets of mitochondrial Sirt3. Collectively, our findings and previous publications suggest that it is the protein targets of acetylation themselves, rather than the degree of global protein acetylation, that play a critical role in cardiomyopathic phenotypes.

Fourth, there is significant increase in many amino acids and certain TCA intermediates (citrate, aconitate, and isocitrate) in affected LS tissues, including cerebellum/brainstem and heart shown by targeted metabolomics. These are likely due to the impairment of various NAD^+^‐dependent dehydrogenases, such as isocitrate dehydrogenase, of which the impairment may contribute to the accumulation of isocitrate, aconitate, and citrate. Likewise, decreased NAD^+^/NADH in LS may impair NAD^+^‐dependent BCKD, which is responsible for BCAA oxidation, leading to accumulation of BCAAs (Figure 1A). NR supplementation significantly increased the NAD^+^ levels, increased NAD^+^/NADH, and restored the metabolic derangement in LS cerebellum/brainstem and heart. Interestingly, several amino acids, particularly BCAAs, are potent inducers of the mechanistic target of the rapamycin (mTOR) signalling pathway, which has been shown to be activated in the LS brain, in concert with increased amino acids in the brainstem.^34^ Rapamycin, a specific inhibitor of mTOR, robustly enhances survival and attenuates disease progression in LS. For example, it delays the onset of neurological symptoms, reduces neuroinflammation, and prevents brain lesions in LS mice.^35^ A recent study reported that rapamycin restored mitochondrial protein levels and reduced the abundance and activity of multiple PKC isoforms in whole brain lysates of LS mice.^36^ Indeed, in a small clinical study, the mitochondrial disease phenotypes of four kidney transplant patients with mitochondrial disease (MELAS) improved after the immunosuppressant was replaced with rapamycin to achieve mTOR inhibition.^37^ However, because rapamycin is an immunosuppressant, the application to treat mitochondrial diseases might be limited by its side effects.

A few treatment options have been proposed to alleviate LS pathologies in the same mouse model used in this study. Apart from rapamycin, chronic hypoxia has been shown to improve survival, body weight, body temperature, behaviour, neuropathology, disease biomarkers, and brain NAD^+^ concentration^38^ in LS mice. In these studies, chronic hypoxia was achieved by putting the LS mice in a hypoxic chamber^39^ or inducing severe anemia by phlebotomy or inhaling low‐dose carbon monoxide.^40^ Although these proof‐of‐concept approaches are interesting, the clinical translation of these therapeutics may be challenging. A recent study using nicotinamide mononucleotide, NMN, also showed that this agent extended the lifespan of Ndufs4^flox/flox^ Meox2‐Cre mice by restoring the NAD^+^ redox balance and lowering the accumulation of hypoxia‐inducible factor 1‐alpha in the skeletal muscle of Ndufs4 KO mice.^41^ However, no apparent beneficial effects were observed in the brain. Our current study elucidates the mechanism underlying the NR‐mediated improvement of encephalopathy in Ndufs4KO mice, that is, deacetylation of p53 to inactivate the p53‐dependent apoptotic pathway and restoration of neuronal GSH, leading to attenuation of neuronal apoptosis and the reduction of microglial activation in the cerebellum and the brainstem region.

The current study is the first to apply human iPS‐CMs and iPS‐Ns to model LS cardio‐encephalomyopathy to elucidate mechanisms responsible for arrhythmia, diastolic dysfunction, and neuronal apoptosis. Using Ndufs4 KO iPS‐CMs, we show that *I*_Na_ and SERCA2a function is decreased in these cells, and that these effects can be prevented by NR treatment. In iPS‐Ns, Ndufs4 deficiency led to hyperacetylation of p53, resulting in increased neuronal apoptosis. The application of iPS modelling to human diseases has great potential to achieve the translation to personalised medicine and test the patient‐specific efficacy of novel therapeutics. Given that NR is a vitamin B3 supplement with excellent safety profiles,^42^ our study provides robust evidence to support the potential clinical use of NR in LS patients.

## 4 MATERIALS AND METHODS

### 4.1 Study design

This study was designed to identify the mechanisms underlying cardio‐encephalomyopathy in LS mice (germline Ndufs4^−/−^deletion). Our hypothesis was tested both in vitro and in vivo using mice and human iPS cells modelling LS. Mice‐included germline Ndufs4^−/−^ (LS mice) and conduction tissue‐specific Ndufs4^−/−^ (driven by HCN4‐CRE). Six to 12 mice in each group were used to achieve a power of 0.8, with significance defined as *p* < .05. The sample size per experiment is included in each figure legend. 21–30‐days‐old Ndufs4^−/−^ mice of both genders were given daily intraperitoneal NR injection (500 mg/kg/day) for 3–4 weeks. We used excessive dose of NR to achieve substantial increase in intracellular NAD^+^ in multiple tissues, as previously reported.^10^ Intraperitoneal route is preferred, since oral intake decreases as neurological symptoms progress in LS mice. For the in vitro studies, we used human embryonic kidney 293 (HEK293) cells and human iPS cells generated from a healthy Caucasian male (ATCC‐1026) followed by deletion of Ndufs4 using the CRISPR/Cas9 method.

### 4.2 Animal models

Germline Ndufs4^−/−^ mice were obtained from the University of Washington.^2^ The HCN4‐Ndufs4 mice were generated by crossing Ndufs4^flox/flox^ mice with Hcn4tm2.1 (cre/ERT2) Sev/J mice (provided by Dr. Ivan Moskowitz, University of Chicago, available at JAX, # 024283 | HCN4CreERT2). Tamoxifen (75 mg/kg, ip) in corn oil was injected daily for 5 days to Ndufs4^flox/flox^ HCN4CreERT2 mice at around 8–10 weeks. All mice were on the C57/BL6/J background and were fed the regular diet from Harlan Teklad. All animal experiments were approved by the Institutional Animal Care and Use Committee (IACUC) at the University of Iowa.

### 4.3 Cell culture

HEK293 cells were obtained from the American Type Culture Collection (ATCC, Manassas, VA) and were cultured in 10% fetal bovine serum (FBS) and 1% PenStrep‐supplemented Dulbecco's Modified Eagle Medium (DMEM) medium. Sirt1 SiRNA (siRNA ID.136459, Catalog #AM16708, ThermoFisher) was used to transfect 293 cells. Briefly, Sirt1 SiRNA was diluted to 100 nM in serum‐free DMEM and Lipofectamine 2000 (Invitrogen) and was incubated at room temperature for 15 min. Cells were transfected for 6–8 h and used for subsequent experiments within 48 h.

### 4.4 Generation of Ndufs knock‐out cells using the CRISPR/Cas9 method

HEK293 cells were cultured to 70%–80% confluence in 6‐cm dishes. Cells were transfected with 2.5 μg of NF2SgRNA or NF4SgRNA (Addgene) and 5 μl of Lipofectamine 2000 (Invitrogen). Forty‐eight hours after transfection, the cells were treated with the appropriate drug and selection was carried out for ∼7 days. The cells were then dissociated to single cells. After cells were counted, they were diluted serially in DMEM medium + 10% FBS to a final concentration of 5 cells per one well of 96‐well plates and expanded for 2–3 weeks. Visible colonies were picked and reseeded in new wells for monolayer growth. Positive candidates were validated by western blotting.

### 4.5 Maintenance of human iPS cells and differentiation of cardiomyocytes and neurons

Human iPS cells were cultured on Matrigel‐coated plates (Corning, Life Sciences) and fed with a mixture of SFM and mTeSR^+^ at a ratio of 75%:25%. For cardiomyocyte differentiation, human iPS cells were passaged using gentle cell dissociation reagent (STEMCELL, catalog #07174) and replated at a density of 3.5 × 10^5^ cells/well on 12‐well plates. Using STEMdiff™ Cardiomyocyte Differentiation and Maintenance kits (STEMCELL, catalog #05010 & #05020), after cells reached 95% confluency, differentiation was initiated by replacing mTeSR with STEMdiff™ Cardiomyocyte Differentiation medium supplemented with Matrigel and STEMdiff™ Cardiomyocyte Differentiation Supplement A. Subsequently, cells were refed every 2 days, using STEMdiff™ Cardiomyocyte Differentiation Supplement B & C, respectively. At day 8, the medium was changed to complete STEMdiff™ Cardiomyocyte Maintenance Medium, and it was changed every other day for 7 days. Cells typically began beating spontaneously on approximately day 8 after differentiation. Cardiomyocytes were enriched from day 10 of differentiation using lactic acid (sodium l‐lactate, Sigma–Aldrich, USA)^43^ in Maintenance Media. Triiodothyronine (T3) was added from ∼Days 20–24 to enhance cardiomyocytes maturation.

For the generation of neural progenitor cells (NPCs), embryonic body (EB) protocol using STEMdiff neural induction medium and SMADi (STEMdiff™ SMADi Neural Induction Kit, Catalog#05835) was implemented. Briefly, aggrewell™800 plates were prepared for experiments by pre‐treatment of the wells with anti‐adherence rinsing solution. Subsequently, each well was seeded with 3×10^6^ cells of single‐cell suspension. Uniform EBs were observed in the aggrewell™800 plates on day 1, ∼75% of the medium was replaced with fresh media every day for 4 days. EBs were harvested and replated at day 5 and medium was fully changed each day for 6 days. The percentage of cells induced to a neuronal fate was calculated on day 8 based on the number of EBs with more than 50% neural rosettes divided by total number of EBs, and it was found to be ∼90%. On Day 12 neural rosettes were selected and replated, and full medium changes were performed daily, using STEMdiff™ Neural Induction medium plus SMADi for another 5 days and the NPCs were passaged on day 18. Neurons were generated from human iPS‐NPCs using the STEMdiff™ Neuron Differentiation kit (STEMCELL, catalog #08500) and STEMdiff™ Neuron Maturation kit (STEMCELL, catalog #08510). Briefly, for neuronal differentiation we used a poly‐l‐ornithine/laminin coating and complete STEMdiff™ Neuron differentiation medium. Neuronal precursors were seeded at a density of 3 × 10^4^ cells/cm^2^ and neurons were allowed to mature for at least 1 week in STEMdiff™ Neuron Maturation medium or Brainphys™ Neuronal medium (STEMCELL, catalog #05790) supplemented with Neurocult™ SM1 Neuronal supplement (STEMCELL, catalog #05711), N2 supplement‐A (STEMCELL, catalog #07152), recombinant human brain derived neurotrophic factor, recombinant human glial‐derived neurotrophic factor, dibutyryl cAMP, and ascorbic acid.

### 4.6 Electrocardiography and transthoracic echocardiograms

ECG recordings were obtained from conscious mice for at least 30 min, using the INDUS Rodent Surgical Monitoring system without anaesthesia. Echocardiograms to measure ventricular size, wall thickness, and ejection fraction were performed on mice using the Vevo 2700 VisualSonics System (Toronto, ON, Canada). Prior to echocardiography, mice were injected with 0.1 mg midazolam, a benzodiazepine with anxiolytic muscle relaxant effects, without any evidence of cardiac suppression. Cardiac images were obtained using a 30‐MHz linear array transducer. Images of the parasternal short and long axis were obtained at a frame rate of ∼180–250 Hz. All image analysis was performed offline using the Vevo 2100 analysis software (v.1.5). Endocardial and epicardial borders were traced on the short axis view during diastole and systole. LV length was measured from endocardial and epicardial borders to the LV outflow tract in diastole and systole. The biplane area–length method was then used to calculate LV mass and ejection fraction. Diastolic function was measured using tissue Doppler imaging of the mitral annulus and conventional mitral inflow (E wave).

### 4.7 Patch‐clamp

Na^+^ current measurements were conducted using the whole‐cell patch‐clamp technique as previously described.^10^ For voltage clamp studies, command pulses were generated using an Axopatch 200B patch clamp amplifier (Molecular Devices, San Jose, CA) and currents were sampled at 20 kHz through an A/D converter (DigiData 1440, Molecular Devices, CA) and low‐pass filtered at 5 kHz. Electrode offset potentials were zero‐adjusted before a Giga‐seal was formed. Fast‐ and slow capacitance was compensated. Approximately 85% series resistance was also compensated, yielding a maximum voltage error of ∼1 mV. To minimise the effects of *I*_Na_ run‐down on the results of the experiments, we carefully monitored the time‐dependent change of *I*_Na_. Recordings were started after the current reached a steady state, normally within 5–8 min. To record Na^+^ currents from HEK293 cells, electrodes of 2–3 MΩ were filled with a pipette solution containing (in mmol/L) NaF 10, CsF 110, CsCl 20, Ethylene glycol‐bis(β‐aminoethyl ether)‐N,N,N′,N′‐tetraacetic acid (EGTA) 10 and HEPES 10 (pH 7.35 with CsOH), and the bath solution contained NaCl 40, 103 NMDG, KCl 4.5, CaCl_2_ 1.5, MgCl_2_ 1, and HEPES 10 (pH 7.35 with CsOH). For recording from human iPS‐CMs, the bath solution contained 50 NaCl, 110 CsCl, 1.8 CaCl_2_, 1 MgCl_2_, 10 HEPES, 10 glucose, 0.0001 CdCl_2_ (pH 7.4 with CsOH). Patch pipettes were filled with 10 NaCl, 135 CsCl, 2 CaCl_2_, 3 MgATP, 2 TEA‐Cl, 5 EGTA and 10 HEPES (pH 7.2 with CsOH). All experiments were performed at room temperature (20–22°C). Current–voltage (*I*–*V*) relationships were generated by plotting the current density elicited by voltage steps from ‐80 mV to +50 mV, at 5 mV intervals, from holding potential of −120 mV. Cellular capacitance was measured, and *I*_Na_ was normalised to cellular capacitance.

### 4.8 Measurement of Ca^2+^‐transient using confocal microscopy

Dissection of the SAN was performed in Tyrode solution (36°C) under the dissecting microscope. The SAN was delineated by the crista terminalis, the orifice of superior vena cava, and the interatrial septum. Spontaneous beating Ca^2+^ or field‐stimulated steady‐state Ca^2+^ transients were measured in non‐patched SAN cells or human iPS‐CMs at ∼35°C. These cells were loaded with Rhod‐2 AM (AAT Bioquest, Inc, Catalog #21062) at room temperature for 30 min, followed by de‐esterification in Tyrode's solution (containing 1.8 mM Ca^2+^) for 15 min. Line‐scan confocal images were acquired at a sampling rate of 1.93 ms per line using a 63×, 1.3 NA oil immersion objective mounted on a Zeiss LSM 510 confocal microscope (Carl Zeiss MicroImaging GmbH). Steady‐state Ca^2+^ transients were achieved by a 30‐s pacing at spontaneous beating for the SAN at 0.5 Hz for human iPS‐CMs. SR Ca^2+^ content was determined after steady‐state stimulation at 1 Hz by measuring the amplitude of the Ca^2+^ release induced by local delivery of 20 mM caffeine. All digital images were processed using IDL 8.0 (Research System Inc).

### 4.9 Immunoprecipitation and immunoblotting

Immunoprecipitation was carried out by incubating 4 μl of the acetyl‐lysine antibody (Cell Signaling Technology, 9814) with 1 mg of tissue homogenate overnight, followed by adding 40 μl of protein A dynabeads (ThermoFisher) for 1 h. After washing, the immunoprecipitates were boiled in SDS‐PAGE gel loading buffer, along with 500 μg of whole‐cell lysates subjected to SDS‐PAGE, transferred to nitrocellulose membrane and probed with a 1:500 dilution of the specified primary antibody and a 1:5000 dilution of peroxidase‐conjugated secondary antibody.

Heart and cerebellar tissues were homogenised in RIPA buffer containing protease inhibitor cocktail (Roche). After quantification with BCA, equal amounts of proteins were loaded onto an SDS‐PAGE gel, followed by standard immunoblotting. Chemiluminescent signal was developed using SuperSignal West Femto Maximum Sensitivity substrate (ThermoFisher, 34095), and blots were imaged using a GelDoc 2000 Chemi Doc system. Band densities were quantified using Image J (NIH).

### 4.10 Antibodies

Primary antibodies used were against Na_V_1.5 (Alomone Labs, ASC‐005), SERCA2a (Proteintech, 67248‐1‐Ig), custom designed anti‐acetyl‐K1479 Na_V_1.5 (YenZym Antibodies), Ndufs2 (Invitrogen, PA5‐22364), Ndufs4 (ABclonal, A13519), p53 (ABclonal, A0263), Acetyl‐p53 (Lys382) (Invitrogen, 710294), Anti‐p53 (acetyl K120) (Abcam, ab78316), cleaved caspase‐3 (Cell Signaling Technology, Asp175), VGluT1(Sigma–Aldrich, ZRB2374), VGAT (Sigma–Aldrich, AMAB91043), and Sirt1 (Abclonal, A11267).

### 4.11 Plasmids

Human Na_V_1.5 (NM_198056.3) was cloned into pcDNA3.1, followed by an internal ribosome entry site (IRES) and the green fluorescent‐protein (GFP) sequence between EcoRI and NotI sites. Mutations of Na_V_1.5 were generated by site‐directed mutagenesis using the Quik‐Change II XL kit (Agilent Technologies, Santa Clara, CA).

### 4.12 Live cell staining and immunostaining

The HEK293 and iPS‐CMs were plated on glass‐bottom dishes. For live staining, the culture medium in the dish was exchanged with prewarmed (37°C) culture medium containing DCFDA (5 mM) and TMRE (25 nM), incubated for ∼30 min, then counterstained with Hoechst 33342 in new medium. For immunostaining, HEK293, iPS‐CM and iPS‐N cells were fixed in 4% paraformaldehyde, blocked, and incubated in primary antibodies overnight. They were then probed with secondary antibody and counterstained with DAPI. Images were acquired using a Leica SP8 confocal microscope. Intensity of fluorescence staining was quantified using ImageJ software.

### 4.13 Metabolic chamber measurements

For analysis of whole‐animal energy expenditure, animals were placed in the Oxymax CLAMS (Comprehensive Lab Animal Monitoring System, Columbus Instruments, Columbus, OH, USA) instruments. These cages have an open circuit system that directly measures various parameters over a 72‐h period, such as heat production, food intake, and movement.^44^ The Oxymax system has an open‐circuit indirect calorimeter for lab animal research, allowing the measurement of oxygen consumption (VO_2_), RER, and activity levels of mice. VO_2_ is a measure of the volume of oxygen used to convert energy substrates into ATP. The RER is calculated as the ratio of carbon dioxide production (VCO_2_) to oxygen consumption and can be used to estimate the fuel source for energy production based on the difference in the number of oxygen molecules required for the oxidation of glucose versus fatty acids. An RER of 0.7 indicates that fatty acids are the primary substrates for oxidative metabolism, whereas an RER of 1.0 indicates that carbohydrates are the primary energy substrates. The locomotor activity was evaluated by the *xy*‐axis detection of animal motion, measured by IR photocell technology in CLAMS metabolic caging system (Columbus Instruments, Columbus, OH). Interruption of an IR beam will accrue one “count” as well as identifying animal position within the respective axis.^45^ All protocols were approved by the University of Iowa Animal Care and Use Committee (IACUC). Heat production was calculated using the equation derived from Lusk (∼1928) to estimate aerobic respiration (heat = 1.232×VCO_2_+3.815×VO_2_). VCO_2_ is described as the rate of carbon dioxide produced by the mouse, and VO_2_ is described as the rate at which oxygen is consumed by the mouse.

### 4.14 Tissue collection for metabolomics assays

All three groups of mice (WT, Ndufs4^−/−^, Ndufs4^−/−^+NR; *n* = 4 each group) were euthanised and dissection was performed on dry ice. The cerebellum/midbrain and heart were rapidly frozen in liquid nitrogen within 1 min of euthanasia.

### 4.15 Metabolomics measurements by GC–MS method

For metabolite extraction, the samples were extracted in ice cold 2:2:1 methanol/acetonitrile/water which contained a mixture of 9 internal standards (d_4_‐Citric Acid, ^13^C_5_‐Glutamine, ^13^C_5_‐Glutamic Acid, ^13^C_6_‐Lysine, ^13^C_5_‐Methionine, ^13^C_3_‐Serine, d_4_‐Succinic Acid, ^13^C_11_‐Tryptophan, d_8_‐Valine; Cambridge Isotope Laboratories) at a concentration of 1 ug/ml each.  The ratio of extraction solvent to sample volume was 18:1. Tissue samples were lyophilised overnight prior to extraction. Tissues were homogenised using a ceramic bead mill homogeniser, after the addition of extraction buffer. The samples were then incubated at −20°C for 1 h followed by a 10‐min centrifugation at maximum speed. Supernatants were transferred to fresh tubes.  Pooled quality control (QC) samples were prepared by adding an equal volume of each sample to a fresh 1.5 ml microcentrifuge tube.  Processing blanks were utilised by adding extraction solvent to microcentrifuge tubes.  Samples, pooled QCs, and processing blanks were evaporated using a speed‐vac. The resulting dried extracts were derivatised using methyoxyamine hydrochloride (MOX) and N,O‐Bis(trimethylsilyl)trifluoroacetamide (TMS) [both purchased from Sigma].  Briefly, dried extracts were reconstituted in 30 μl of 11.4 mg/ml MOC in anhydrous pyridine (VWR), vortexed for 10 min, and heated for 1 h at 60°C. Next, 20 μl TMS was added to each sample, and samples were vortexed for 1 min before heating for 30 min at 60°C. The derivatised samples, blanks, and pooled QCs were then immediately analysed using GC/MS.

GC chromatographic separation was conducted on a Thermo Trace 1300 GC with a TraceGold TG‐5SilMS column (0.25 μm film thickness; 0.25 mm ID; 30 m length). The injection volume of 1 μl was used for all samples, blanks, and QCs. The GC was operated in split mode with the following settings: 20:1 split ratio; split flow: 24 μl/min, purge flow: 5 ml/min, carrier mode: constant flow, carrier flow rate: 1.2 ml/min). The GC inlet temperature was 250°C.  The GC oven temperature gradient was as follows: 80°C for 3 min, ramped at 20°C/min to a maximum temperature of 280°C, which was held for 8 min. The injection syringe was washed three times with pyridine between each sample. Metabolites were detected using a Thermo ISQ single quadrupole mass spectrometer. The data were acquired from 3.90 to 21.00 min in EI mode (70 eV) by single ion monitoring (SIM). Metabolite profiling data were analysed using Tracefinder 4.1 utilising standard verified peaks and retention times.

We used TraceFinder 4.1 to identify metabolites in extracted samples, blank, and QCs. We do this by comparing sample metabolite peaks against an in‐house library of standards. The standard library was prepared by processing and analysing authentic standards via the method described above.  We created a database of retention times and three fragment ions for each metabolite standard: a target peak/ion and two confirming peaks/ions. When running biological samples, metabolites are identified that not only match with the known retention times of the authentic standard, but also with its target and confirming peaks. TraceFinder was also used for gas chromatography‐mass spectrometry (GC–MS) peak integration to obtain peak areas for each metabolite. After TraceFinder analysis, instrument drift over time is corrected for using local regression analysis as described by Li et al.^46^ Pooled QC samples which were run in duplicate at the beginning and end of the GC–MS run were used for this purpose. The data are then normalised to an internal standard to control for extraction, derivatisation, and/or loading effects.

### 4.16 Sample preparation for LC–MS

For metabolite extraction, samples were extracted in ice cold 2:2:1 methanol/acetonitrile/water which contained a mixture of 9 internal standards (d4‐citric acid, 13C5‐glutamine, 13C5‐glutamic acid, 13C6‐lysine, 13C5‐methionine, 13C3‐serine, d4‐succinic acid, 13C11‐tryptophan, d8‐valine; Cambridge Isotope Laboratories) at a concentration of 1 μg/ml each.  The ratio of extraction solvent to sample volume was 18:1. Tissue samples were lyophilised overnight prior to extraction. Tissues were homogenised using a ceramic bead mill homogeniser, after the addition of extraction buffer. Samples were then incubated at −20°C for 1 h followed by a 10 min centrifugation at maximum speed. Four hundred microliters of supernatants were transferred to fresh tubes.  Pooled QC samples were prepared by adding an equal volume of each sample to a fresh 1.5 ml microcentrifuge tube. Processing blanks were utilised by adding extraction solvent to microcentrifuge tubes. Samples, pooled QCs, and processing blanks were evaporated using a speed‐vac. The resulting dried extracts were reconstituted in 40 μl of acetonitrile/water (1:1, V/V), vortexed and samples, blanks, and pooled QCs were then analysed using liquid chromatographymass spectrometry (LC–MS).

### 4.17 LC–MS Analysis

Two microliters of metabolite extracts were separated using a Millipore SeQuant ZIC‐pHILIC (2.1 × 150 mm, 5 μm particle size) column with a ZIC‐pHILIC guard column (20 × 2.1 mm) attached to a Thermo Vanquish Flex UHPLC. Mobile phase comprised Buffer A—20 mM (NH_4_)2CO_3_, 0.1% NH_4_OH and Buffer B: acetonitrile. The chromatographic gradient was run at a flow rate of 0.150 ml/min as follows: 0–21 min‐linear gradient from 80% to 20% Buffer B; 20–20.5 min‐linear gradient from 20% to 80% Buffer B; and 20.5–28 min‐hold at 80% Buffer B. Data were acquired using a Thermo Q Exactive mass spectrometer operated in full‐scan, polarity‐switching mode with a spray voltage set to 3.0 kV, the heated capillary held at 275°C, and the HESI probe held at 350°C. The sheath gas flow was set to 40 units, the auxiliary gas flow was set to 15 units, and the sweep gas flow was set to 1 unit. MS data acquisition was performed in a range of *m*/*z* 70–1,000, with the resolution set at 70,000, the AGC target at 10^e6^, and the maximum injection time at 200 ms. Acquired LC–MS data were processed by Thermo Scientific TraceFinder 4.1 software, and metabolites were identified based on the University of Iowa Metabolomics Core facility in‐house, physical standard‐generated library. NOREVA was used for signal drift correction.^40^ Data per sample were then normalised to an internal standard (13C5‐Methionine) to control for extraction, derivatisation, and/or loading sample effects.

### 4.18 Citrate synthase assay

Citrate synthase enzyme activity was measured as previously described.^47^ Briefly, cell lysates was homogenised in a buffer containing 250 mM sucrose, 20 mM tris, 40 mM KCl, and 2 mM EGTA at a pH of 7.4. CS reaction was performed with 5 μg protein lysate in a final solution of 200 μl containing 200 mM tris with a pH of 8.0, 0.2% v/v Triton‐X‐100, 100 μl of 5,5′‐dithiobis (2‐nitrobenzoic acid) (DTNB), 10 mM acetyl‐CoA, and 0.5 mM oxaloacetic acid, and the reaction was monitored at 412 nm for 3 min with a spectrophotometer.

### 4.19 Gene expression analysis

RNA was purified from cerebellum lysates homogenized in RNA Protection Reagent using Monarch Total RNA Miniprep Kit (Biolabs, NEB #T2010) and reverse transcribed. Real‐time qPCR analyses were performed on a QuantStudio™ 3 real‐time PCR system (Applied Biosystems™) using Taqman mastermix. Primers used for gene expression, *Aen* (NM_001162939(2)), *Bax* (NM_007527(1)), *Traf4* (NM_009423(1)) were purchased from IDT (PrimeTime qPCR Probe Assays). Transcripts were quantified using the ΔΔ*Ct* method and were normalised to 18s rRNA gene expression.

### 4.20 Measurement of SIRT1 activity

SIRT1 activity was measured in the nuclear extract from heart or cerebellum tissues using the SIRT1 Activity Assay Kit (ab156065, Abcam) according to the manufacturer's instructions.  Fluorescence intensity was measured for 60 min at 2 min intervals on a microplate fluorometer (excitation, 350 nm; emission, 460 nm). SIRT1 activity was calculated within the linear range of reaction velocity and normalised against the protein concentration in WT control extracts.

### 4.21 Statistics

All analyses and calculations were performed using GraphPad Prism (version 8.0; GraphPad Software, Inc., CA, USA) and/or Stata IC version 10. The results were expressed as the mean ± standard error of the mean for normally distributed data or proportion for categorical data. Differences between groups were evaluated using one‐way or two‐way analysis of variance (ANOVA) in multiple groups’ comparison, with Sidak or Tukey post hoc tests for comparison between two groups after ANOVA. Student's *t*‐test was used when only two groups were compared. Skewed data are analysed using non‐parametric tests, including Kruskal–Wallis test (for multiple groups) or Mann–Whitney (for two groups’ comparison). The statistical methods were summarised in each figure legend. Survival curves were analysed using Kaplan–Meier method, followed by log‐rank test to test for significant difference between groups. Metabolomics data were analysed using ANOVA (three‐group comparison), followed by Sidak post hoc for two‐group comparisons. To control for false discovery rate (FDR) in metabolomics multiple testing, we used Michael Anderson's code to compute the *q*‐values^48^ by Stata (now listed in Tables S2 and S3). The FDR is the expected proportion of rejections that are type I errors (false rejections).

## CONFLICT OF INTEREST

The authors declare that there is no conflict of interest that could be perceived as prejudicing the impartiality of the research reported.

## AUTHOR CONTRIBUTIONS

JYY, ND, DFD performed experiments, data analysis and manuscript writing; YC, BC performed experiments and assisted in data analysis; MH performed neuropathological analysis; VA provided reagents; KI, LSS, BL, EDA and CB supervised the study, provided reagents and provided critical revision and editing of the manuscript; DFD developed the concept and design of the study.

## Supporting information



Supplementary material

Supplementary material

Supplementary material

Supplementary material

Supplementary material

Supplementary material

Supplementary material

Supplementary material

Supplementary material
