# Supplementary material for: Metabolic rescue ameliorates mitochondrial encephalo‐cardiomyopathy in murine and human iPSC models of Leigh syndrome
Source: Clin Transl Med. 2022 Jul 25;12(7):e954. doi: 10.1002/ctm2.954 (PMC9309541; doi:10.1002/ctm2.954)
Supplement: Supplementary file 8 — Supplementary material [file CTM2-12-e954-s004.docx]

**Supplemental Figure legend**

**Figure S1. Body weight, heart weight, ventricle size and fibrosis of WT and Ndufs^-/-^ mouse.** (**a**) LS mice were runted with the average body weight ~60% of wild type (WT) littermates and post-weaning NR supplementation did not alter this. (**b**) Heart weight and (**c**) normalized heart weight was not significantly different from that in WT mice and this was not affected by NR. (**d-f**) Trichrome staining of left ventricles; (**g**) Quantitative analysis of trichrome blue area; *p<0.05 compared with WT by Sidak post-hoc analysis. n=5-7.

**Figure S2. (a)** Ca^2+^-transients from SA nodes isolated from three Ndufs4^-/-^ (LS) mice showing spectrum of sinus node dysfunction, including intermittent sinus pauses and bradycardia, *upper*; marked bradycardia with episodic tachycardia (bradycardia-tachycardia), *middle*; and marked sinus bradycardia, *lower.* (**b**) Ndufs4^-/-^ mice developed various forms of cardiac arrhythmia. Normal sinus rhythm in HCN4-Cre injected with tamoxifen (n=3), *upper*; premature contractions in 4 of 8 (50%) HCN-4-specific Ndufs4^-/-^ mice, including APC, atrial arrhythmia and/or VPC; an example of VPCs is shown (arrows), *middle*; sinus node dysfunction (arrows) and bradyarrhythmia (dotted line) was recorded in an additional 3 of 8 (37.5%) HCN4-Ndufs4^-/-^ mice. One HCN4-Ndufs4^-/-^ mouse (12.5%) showed normal sinus rhythm, *bottom.*

**Figure S3. Effect of Ndufs4 KO and NR on acetylation of K1479 in Na_V_1.5 in heart tissues and HEK cells.** (**a-c**) Immunohistochemistry staining of K1479 in Na_V_1.5 in left ventricular sections of WT and Ndufs4^-/-^  mice untreated or treated with NR (Scale bar: 50um). Representative immunoblotting of (**d**) total acetyl-lysine (**e**) Acetyl-Na_V_1.5 (K1479) and (**f**) total Na_V_1.5 in the heart tissue lysates of WT and Ndufs4^-/-^  mice untreated or treated with NR (n=3 per group).

**Figure S4. (a)** Immunoblotting of total lysine acetylation in HEK293 and Ndufs2 knockout cells (n=3). (**b**) Citrate synthase in HEK293(WT), Ndufs4KO and Ndufs2KO cell lines (n=3). (**c-d**) Live staining of Ndufs2KO and Ndufs2KO with Sirt1 knockdown (Sirt1 siRNA) cells untreated or treated with NR with markers of mitochondrial membrane potential (TMRE), total cellular ROS (DCFDA) and Hoechst (blue) and quantification (n=5-6 per group). Data are mean ± s.e.m. Statistical significance was determined by ANOVA and post-hoc analysis.

**Figure S5. Sirt-1 mediated acetylation of K1479-Na_V_1.5 is responsible for the effect of Ndufs2 KO or NR supplementation on I_Na_.** Normalized current–voltage relationships of K1479-Na_V_1.5 or mutant Na_V_1.5 constructs mimicking acetylation (K1479Q) expressed in (**a**) wild-type HEK293 cells or in (**c**) Ndufs2KO HEK293 cells. (**b, d**) Relative I_Na_ density at –25 mV from data in panel *a* and *c* respectively. (**e**) Normalized current-voltage relationships of K1479-Na_V_1.5 or deacetylation mimic K1479R-Na_V_1.5 expressed in wild-type vs. Ndufs2KO HEK 293 cells. (**f**) Relative I_Na_ density at –25 mV from data in panel *e*. (**g-h**) Normalized current-voltage relationships and relative I_Na_ density at –25 mV of wild-type HEK293 cells expressing K1479-Na_V_1.5 (control, vehicle) or treated with 5 mM NR with or without Sirt1 selective inhibitor Ex-527 (5 mM) for 48 hr. Data are mean ± s.e.m. of biologically independent samples. *p<0.05, NS: not significant, by ANOVA test.

**Figure S6. SIRT1 activity in mouse heart and brain extracts.** SIRT1 activity in heart (**a**) and cerebellum (**b**) extracts from WT or Ndufs4^-/-^mice. The mice were administered with the vehicle or NR as indicated. Data are mean ± s.e.m. of biologically independent samples.

**Figure S7.** (**a-b**) Representative western blot of K382-p53 and K120-p53 in mouse cerebellum/midbrain from WT, Ndufs4KO and Ndufs4KO treated with NR and (**c**) Quantification (Data are representative of three independent experiments). (**d**) Relative mRNA expression of Aen, Bax and Traf4 from cerebellum of WT, Ndufs4KO and Ndufs4KO treated with NR (n=5-6 per group) (**e**) Survival curve of Ndufs4^-/-^ mice (n=14-17). Data are mean ± s.e.m. Statistical significance was determined by t-test (*c*) or ANOVA and post-hoc analysis (d). * p<0.05.

**Figure S8**. Immunohistochemistry of mouse heart ventricles for cleaved caspase-3 apoptotic marker is negative for all three groups: (**a**) WT, (**b**) Ndufs4^-/-^ , and (**c**) Ndufs4^-/-^ + NR; n=3. Immunoblotting of (**d**) acetyl p-53 (K120) and (**e**) Sirt1 in the hearts of WT, Ndufs4KO and Ndufs4KO treated with NR mice (n=3). Data are mean ± s.e.m. Statistical significance was determined by ANOVA. ns not significant.

**Figure S9.** Representative Immunofluorescence staining of iPS-CM at (**a**) Day 25, (**b**) Day 21 post-differentiation showing distribution of Ndufs4 and highlighting striated pattern of Troponin.

**Supplemental Video legends**

**Video S1**. Ndufs4 KO untreated, showing marked decrease in activity, tremor, and cerebellar ataxia (rotational movement)

**Video S2**. Ndufs4 KO treated with NR, showing improvement of activity with the ability to walk straight with milder cerebellar symptoms (tremor and ataxia)

**Video S3**. Ndufs4 KO untreated, showing marked decrease in activity, tremor, and cerebellar ataxia (rotational movement)

**Video S4**. Ndufs4 KO treated with NR, showing improvement of activity with the ability to walk straight with milder cerebellar symptoms (tremor and ataxia)

**Supplemental Table legends**

**Table S1**. Selective Metabolites that Increased or Decreased by at least 20% in Leigh Syndrome Hearts compared with Wild Type Hearts (Pilot Data)

**Table S2**. Cardiac metabolites measured by GC/MS for WT, Leigh Syndrome (LS) and NR-treated LS mice (NR-LS)

**Table S3**. Metabolites from Cerebellum/Brainstem Measured by GC/MS for WT, Leigh Syndrome (LS) and NR-treated LS mice (NR-LS)
